# Integrated Transcriptomic Analysis Reveals Coordinated Gliovascular Remodeling Across Diffuse Glioma Grades

**DOI:** 10.3390/cancers18182908

**Published:** 2026-09-09

**Authors:** JuliAnne E. Allgood, Kaitlyn Miller, Lindsey Burnsed, Jessica E. Pullan

**Affiliations:** 1 College of Osteopathic Medicine, Rocky Vista University, Ivins, UT 84738, USA; 2 Department of Chemistry and Physics, Southern Utah University, Cedar City, UT 84720, USA; jessicapullan@suu.edu

**Keywords:** gliovascular unit, bioinformatics, glioma, glymphatics, astrocyte, AQP4

## Abstract

**Background:** The gliovascular unit forms the structural and functional basis of the glymphatic system, which facilitates cerebrospinal fluid–interstitial fluid exchange to maintain brain homeostasis. Although disruption of individual astrocytic, vascular, and extracellular matrix (ECM) components has been described in diffuse glioma, it remains unknown whether these alterations represent isolated pathological events or a coordinated remodeling program across glioma grades. **Methods:** Bulk transcriptomic signatures representing eight components of the gliovascular unit were quantified in The Cancer Genome Atlas (TCGA) and independently validated in the Chinese Glioma Genome Atlas (CGGA). Pathway activity was compared across WHO grades and IDH status, followed by gene-level, network, and grade-specific analyses to characterize the grade-associated patterns of gliovascular remodeling. Laser-capture microdissected RNA sequencing data from the Ivy Glioblastoma Atlas Project was subsequently used to define the anatomical distribution of representative gliovascular markers within glioblastoma (GBM). Finally, multivariable Cox proportional hazards regression was performed to evaluate associations between gliovascular pathway activity and overall survival. **Results:** Diffuse glioma was characterized by coordinated grade-associated remodeling of the gliovascular unit across independent patient cohorts. Lower-grade tumors were characterized by greater expression of homeostatic astrocyte- and endfoot-associated transcriptional programs, whereas higher-grade tumors showed increasing inflammatory, ECM-remodeling, basement membrane-remodeling, and angiogenic signatures. Regional analysis demonstrated corresponding anatomical compartmentalization of these transcriptional features within GBM. Despite closely reflecting glioma grade, gliovascular remodeling was not independently associated with overall survival. **Conclusions:** Coordinated grade-associated remodeling of the gliovascular unit provides a unifying framework linking astrocyte-associated, inflammatory, and vascular remodeling transcriptional programs across glioma grades. This systems-level model establishes a foundation for future mechanistic studies examining how disruption of gliovascular homeostasis contributes to glymphatic dysfunction, sleep disturbance, and disease progression.

## Simple Summary

Gliomas are brain tumors that disrupt the normal relationship between brain cells, blood vessels, and the surrounding tissue. It is not well understood whether these disruptions occur alone or as part of a coordinated process across increasing tumor grades. We examined gene activity in publicly available glioma data from real patients to determine how this system differs across tumor grades. We identified a consistent pattern in which normal supportive functions were lower in higher-grade tumors, while inflammation, tissue remodeling, and abnormal blood vessel growth became increasingly prominent. Our findings provide a new framework for understanding how the environment surrounding glioma cells is organized across glioma grades and guide future research on how disruption of normal brain fluid transport and vascular function contributes to glioma biology.

## 1. Introduction

The glymphatic system is a brain-wide fluid transport and waste-clearance pathway in which cerebrospinal fluid (CSF) enters along perivascular spaces, exchanges with interstitial fluid (ISF), and facilitates removal of metabolites from the brain parenchyma [1,2]. This process is supported by the close anatomical relationship between cerebral vessels and perivascular astrocyte endfeet. Polarized aquaporin-4 (AQP4) channels located on astrocyte endfeet have been implicated in CSF–ISF exchange, although the precise mechanisms governing glymphatic transport remain debated [3]. These perivascular structures exist within a broader neurovascular architecture in which astrocytes, endothelial cells, pericytes, basement membrane (BM), and other cellular and extracellular components interact to maintain vascular integrity and brain homeostasis [4,5]. Together, these interconnected elements form the gliovascular unit, an interface that provides the structural foundation for perivascular fluid transport and links glymphatic function to the integrity of the surrounding vascular and astrocytic microenvironment.

Glymphatic dysfunction is increasingly implicated in glioma biology, where impaired perivascular transport may contribute to waste retention, altered ISF dynamics, and an inflammatory and immunosuppressive tumor microenvironment (TME) [6]. Experimental imaging studies have demonstrated reduced glymphatic influx and clearance in glioblastoma (GBM) models [7], while human imaging studies have associated increasing tumor burden with impaired glymphatic clearance. This literature supports a relationship between malignant progression and disruption of perivascular fluid transport [8]. These changes occur alongside characteristic features of the GBM microenvironment, including hypoxia, immune-cell infiltration, blood–brain barrier (BBB) disruption, and altered astrocyte–vascular interactions [9,10]. Disruption of astrocytic endfeet, AQP4 organization, and the basal lamina may further compromise the structural relationship between astrocytes and the cerebral vasculature [11]. Moreover, tumor-associated astrocytes can transition toward reactive phenotypes through signaling pathways including JAK/STAT, contributing to altered inflammatory signaling and tumor–immune interactions [12]. Together, these observations suggest that impaired glymphatic transport in GBM occurs within broader remodeling of the cellular and extracellular architecture that normally supports gliovascular homeostasis.

Diffuse glioma provides a biological continuum for examining how disruption of the gliovascular interface changes with increasing malignancy. WHO grade and isocitrate dehydrogenase (IDH) status capture clinically and biologically distinct tumor phenotypes and provide complementary measures for evaluating gliovascular alterations associated with disease aggressiveness [13,14]. Emerging imaging evidence further suggests that glymphatic dysfunction varies with glioma molecular characteristics, including IDH mutation status, indicating that impaired brain fluid transport may be linked to underlying tumor biology rather than simply tumor presence [15]. Examining gliovascular pathways across these disease states may therefore distinguish alterations that emerge early from those associated with advanced malignant progression and reveal relationships that are not apparent when individual components of the gliovascular interface are studied independently.

Previous studies have characterized individual components of the glioma microenvironment, including tumor-associated reactive astrocytes [16], extracellular matrix (ECM) remodeling [17,18], and tumor-associated macrophage and inflammatory programs [19,20]. Collectively, this work demonstrates extensive cellular and extracellular remodeling in glioma, but these processes have largely been investigated as distinct features of tumor biology. How these alterations relate to one another and become organized across diffuse glioma progression therefore remains incompletely understood. A systems-level analysis of the gliovascular unit may reveal whether they represent independent consequences of malignancy or components of a coordinated remodeling process.

Here, we characterized transcriptional programs representing eight components of the gliovascular unit across two independent diffuse glioma cohorts to determine whether their disruption occurs independently or as part of a coordinated remodeling process. Pathway- and gene-level analyses were integrated with network and transition-specific modeling to define the organization of gliovascular remodeling across malignant progression, while regional Ivy GAP analysis was used to examine how representative features of this remodeling are distributed within the GBM microenvironment. We further evaluated whether gliovascular pathway activity was independently associated with overall survival beyond established clinical variables. Together, these analyses establish a systems-level framework for investigating how the molecular architecture supporting normal gliovascular homeostasis is remodeled during diffuse glioma progression.

## 2. Materials and Methods

### 2.1. Patient Cohorts and Data Sources

Bulk RNA sequencing data and matched clinical annotations were obtained from two independent glioma cohorts: The Cancer Genome Atlas (TCGA; *n* = 800) [21] and the Chinese Glioma Genome Atlas (CGGA; *n* = 422) [22]. TCGA served as the discovery cohort, while CGGA was used for independent replication. The TCGA expression dataset was obtained using TCGAbiolinks R package from the TCGA low (LGG)- and high-grade glioma (HGG) projects. RNA-sequencing expression data were obtained from the GDC STAR-Counts workflow using upper-quartile normalized fragments per kilobase of transcript per million mapped reads (FPKM-UQ). The combined TCGA-LGG/HGG expression dataset initially contained 925 RNA-sequencing specimens. Analyses were restricted to primary tumors with available WHO grade (grade II, *n* = 214; grade III, *n* = 241; grade IV, *n* = 345). The CGGA expression dataset was derived from the mRNAseq_693 cohort and initially contained 693 samples; analyses were restricted to primary tumors with available WHO grade, yielding 422 tumors (WHO grade II, *n* = 138; grade III, *n* = 144; grade IV, *n* = 140).

Clinical variables included WHO grade, IDH mutation status, and overall survival. Samples missing information required for a particular analysis were excluded from that analysis. Overall survival for TCGA was derived from the available clinical follow-up fields (vital_status, days_to_death, and days_to_last_follow_up), yielding survival data for 798 specimens. Overall survival data were available for 366 CGGA tumors.

To evaluate the influence of tumor composition, tumor purity estimates generated using the ABSOLUTE algorithm and immune/stromal infiltration estimates generated using the ESTIMATE algorithm were obtained for subsets of the TCGA cohort (*n* = 683 and *n* = 670, respectively). These variables were used exclusively for sensitivity analyses. Equivalent estimates were not available for CGGA. To further assess the cellular specificity of the marker genes used to construct the pathway activity scores, expression of the 55 available marker genes was examined using the public core GBmap single-cell GBM reference comprising 338,564 cells. For each gene, the percentage of cells expressing the transcript and its relative scaled expression were evaluated across annotated malignant, glial, vascular, myeloid, lymphoid, and neuronal cell populations. Results are presented as a dot plot in Figure S5, with dot size representing the percentage of cells expressing each gene and color representing relative row-scaled expression across cell types.

Anatomically resolved regional analysis was performed using the Ivy Glioblastoma Atlas Project (Ivy GAP), which provides laser-capture microdissected RNA-sequencing data from anatomically distinct regions of human IDH wildtype GBM. Expression data were analyzed from the leading edge (LE), infiltrating tumor (IT), cellular tumor (CT), pseudopalisading cells surrounding necrosis (PAN), and microvascular proliferation (MVP). Gene expression matrices, gene annotations, and regional metadata were downloaded from the Ivy GAP portal and integrated for downstream regional analyses.

### 2.2. Gliovascular Unit Activity Scoring

Eight pathway activity scores representing major components of the glymphatic-associated gliovascular unit were generated from curated marker gene panels describing homeostatic astrocytes [23,24], reactive astrocytes [12,16], neuroinflammation [9,19], endothelial angiogenesis [10,11], BM remodeling [3,5,11,25], extracellular matrix (ECM) remodeling [17,18], astrocyte endfoot organization [1,2,3,11], and BBB specialization [3,4,10,26] (Table 1). For each cohort, marker genes present in the expression matrix (minimum of three genes per pathway) were standardized (z-scores) after excluding genes with zero variance. Pathway activity scores were calculated as the mean standardized expression of all available marker genes within each pathway. Gene standardization and pathway scoring were performed independently within each cohort. Fifty-six marker genes were available for scoring TCGA and 55 in CGGA because PECAM1 was unavailable in the CGGA expression matrix.

### 2.3. Association of Pathway and Gene Expression with Clinicopathologic Variables

Associations between gliovascular unit pathway activity and WHO grade were evaluated separately in the TCGA and CGGA cohorts using one-way ANOVA, followed by Tukey’s HSD post hoc test for pairwise comparisons between grades. Robustness of grade-associated pathway effects to the scoring method was evaluated using an alternative ssGSEA-based pathway scoring approach (Table S1). Individual marker genes (56 in TCGA; 55 in CGGA, where PECAM1 was unavailable) were evaluated by comparing standardized expression between WHO grade II and grade IV tumors using Welch’s *t*-test. Standardized expression of the gliovascular unit pathways was also compared by IDH mutation status using two-sample *t*-tests. Group differences at both the pathway and gene levels were visualized using violin plots. Robustness of pathway-level grade associations to individual gene membership was evaluated using a leave-one-gene-out sensitivity analysis for all eight pathways in both cohorts (Table S2).

### 2.4. Association with Neftel-Derived Transcriptional Programs

To assess whether gliovascular-associated transcriptional programs covaried with expression patterns resembling the malignant-cell states described by Neftel et al., pathway scores were correlated using Pearson correlation with bulk transcriptional scores corresponding to astrocyte-like (AC-like), oligodendrocyte progenitor-like (OPC-like), neural progenitor-like (NPC-like), mesenchymal-like (MES-like), and cycling programs. The AC-like and OPC-like scores were derived from the corresponding Neftel meta-modules, whereas NPC-like and MES-like scores incorporated the paired NPC1/NPC2 and MES1/MES2 meta-modules, respectively. Cycling incorporated the G1/S and G2/M programs [27]. Because the Neftel framework was originally defined in IDH-wildtype GBM, these scores were interpreted as bulk transcriptional resemblance to the corresponding programs rather than as direct measures of malignant-cell state abundance. Analyses were performed independently in the TCGA and CGGA cohorts. To assess potential inflation of pathway–program correlations by shared gene membership, gene overlap between the eight gliovascular signatures and the Neftel meta-modules was quantified, and correlations were repeated after removing overlapping genes from the affected gliovascular signatures. *p*-values were adjusted using the Benjamini–Hochberg procedure within each cohort. Results of the gene-overlap sensitivity analysis are provided in Table S3.

### 2.5. Grade-Specific Remodeling and Loss-Versus-Gain Analysis

To determine whether the magnitude of gliovascular pathway differences varied across grade comparisons, Hedges’ g effect sizes (with small-sample correction) were calculated separately for the WHO grade II→III and WHO grade III→IV grade comparisons at both the pathway and individual gene levels. Differences between grade-specific effect sizes (Δg = gIII→IV − gII→III) were used to quantify whether pathway remodeling differed in magnitude between the WHO II→III and WHO III→IV grade comparisons. For the pathway-level analysis, uncertainty in Δg was estimated using 2000 bootstrap iterations. Within each iteration, specimens were resampled with replacement separately within WHO grades II, III, and IV, with the same resampled grade III group used to calculate both effect sizes. Percentile-based 95% confidence intervals were obtained from the bootstrap distribution of Δg, and two-sided bootstrap *p*-values were calculated relative to the null hypothesis Δg = 0. *p*-values were adjusted for multiple testing using the Benjamini–Hochberg procedure across the eight pathways within each cohort.

Marker genes were classified a priori into Loss and Gain categories based on their individual biological function within the gliovascular unit (Table 1) to assess whether gene-level differences in grade-associated effect sizes reflected coordinated biological processes. This classification aligned with whole-pathway membership for homeostatic astrocyte, endfoot organization, endothelial angiogenesis, ECM remodeling, BM, and neuroinflammation genes. Within the reactive astrocyte and BBB specialization pathways, genes were classified individually according to biological role: structural and identity markers (GFAP, GJA1, S100B; CLDN5, OCLN, TJP1) were assigned to the Loss category, while reactive and inflammatory markers (VIM, SERPINA3, LCN2) were assigned to the Gain category. Genes with context-dependent biological directionality (MFSD2A, SLC2A1, and ABCB1) were excluded from the primary analysis. Grade-specific effect sizes were then compared between Loss and Gain gene sets using Mann–Whitney U tests. A sensitivity analysis excluding KCNJ10, the largest-magnitude Loss-category gene, confirmed that the result was not driven by this single gene.

### 2.6. Regional Expression of Gliovascular Genes Across the GBM Microenvironment

Representative sentinel genes identified from the bulk transcriptomic analyses were examined using Ivy GAP. Nine genes representing all eight biological components of the gliovascular unit were selected as representative sentinel genes: KCNJ10 (homeostatic astrocytes), SNTA1 and AQP4 (astrocyte endfoot organization and glymphatic support), SERPINA3 (reactive astrocytes), CCL2 (neuroinflammation), FN1 (ECM remodeling), COL4A1 (vascular BM), ANGPT2 (angiogenesis), and TJP1 (BBB specialization). Gene expression was evaluated across anatomically annotated tumor regions and transformed as log_2_(FPKM + 1) prior to analysis. Regional differences among the LE, IT, CT, PAN, and MVP were evaluated using linear mixed-effects models with anatomical region as a fixed effect and donor tumor as a random intercept to account for repeated sampling within tumors. Overall regional effects were evaluated using Type III ANOVA with Satterthwaite approximation of denominator degrees of freedom. Pairwise regional comparisons were performed using Tukey-adjusted estimated marginal means. Complete mixed-effects-model results and pairwise regional comparisons are provided in Tables S4 and S5, respectively.

Ivy GAP analyses were restricted to the Anatomic Structures RNA-sequencing dataset and to samples designated as reference histology, yielding 122 laser-capture microdissected specimens from 10 unique GBM tumors. Samples represented five anatomically defined regions: LE (*n* = 19 samples from 8 tumors), IT (*n* = 24 from 8 tumors), CT (*n* = 30 from 10 tumors), MVP (*n* = 25 from 9 tumors), and PAN (*n* = 24 from 8 tumors). Regional representation was therefore not perfectly balanced across tumors. The mixed-effects modeling framework accounted for this repeated and unequal regional sampling by including donor tumor as a random intercept.

### 2.7. Grade-Adjusted Pathway Network and Integrative Remodeling Model

To identify coordinated relationships among glymphatic-associated pathways independent of tumor grade, pathway scores were first residualized against WHO grade using linear regression. Pairwise Pearson correlations were then calculated between residualized pathway scores independently in the TCGA and CGGA cohorts, with *p*-values adjusted for multiple testing using the Benjamini–Hochberg procedure. Pairwise Spearman correlations among the unadjusted pathway scores were additionally examined in each cohort to characterize the overall concordance among pathway programs (Table S6). A cross-sectional pathway model was subsequently constructed by retaining only pathway relationships that demonstrated significant partial correlations with consistent directionality in both cohorts. Relationships within the model were organized according to the grade comparison at which each pathway exhibited its greatest relative change based on pathway-level Δg values, providing a hypothesis-generating framework for coordinated gliovascular remodeling across glioma grades.

The robustness of the pathway model was evaluated by accounting for potential confounding from tumor purity and immune infiltration. Grade-adjusted network analyses were repeated after additionally adjusting for ABSOLUTE tumor purity to evaluate the five core pathway relationships (Table S7) and for ESTIMATE immune scores to evaluate the three network edges involving neuroinflammation. Sensitivity analyses were restricted to the TCGA cohort because equivalent purity and immune infiltration estimates were unavailable for CGGA.

### 2.8. Survival Analysis

The prognostic significance of each glymphatic pathway was evaluated using Cox proportional hazards regression. Separate models were fitted for each pathway while adjusting for WHO grade and IDH mutation status in both cohorts. Age was additionally included as a covariate where available. The proportional-hazards assumption was evaluated using Schoenfeld residuals with the cox.zph function in the R survival package. Independent prognostic value was assessed using likelihood-ratio tests comparing models with and without the pathway score, and *p*-values were adjusted for multiple testing using the Benjamini–Hochberg procedure within each cohort.

### 2.9. Statistics

Statistical analyses were performed in R (version 4.5.3) and Python (version 3.12). In R, linear regression, linear mixed-effects modeling, survival analysis, pathway-level grade-specific Hedges’ g and bootstrap analyses, and the Ivy GAP spatial analysis were performed using the stats, lme4, lmerTest, emmeans, and survival packages. In Python, pairwise group comparisons, correlation analyses, the grade-adjusted pathway network, and the Loss-versus-Gain comparison were performed using SciPy, with data handling in pandas and NumPy. Data visualization was performed using ggplot2 in R and Matplotlib in Python. Unless otherwise specified, *p*-values were adjusted for multiple testing using the Benjamini–Hochberg procedure.

## 3. Results

### 3.1. The Gliovascular Unit Demonstrates Coordinated Remodeling Across Glioma Grades

**Figure 1:**
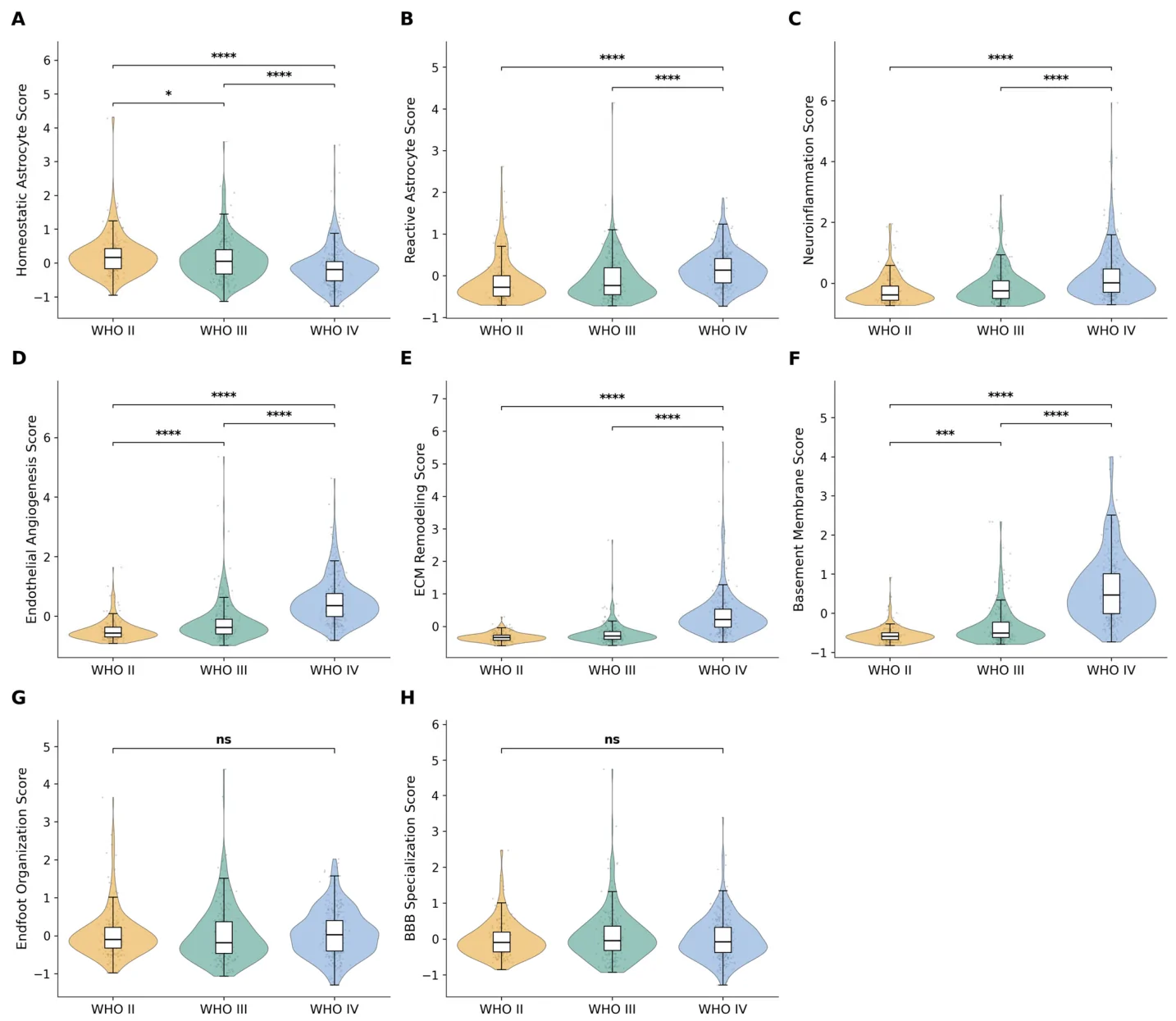
Pathway activity scores across WHO glioma grades, TCGA. Violin plots of eight pathway activity scores ((**A**–**H**): homeostatic astrocytes, reactive astrocytes, neuroinflammation, endothelial angiogenesis, ECM remodeling, BM remodeling, endfoot organization, BBB specialization) stratified by WHO grade (II, III, IV) in TCGA (*n* = 800). Box plots show median and interquartile range. Significance from one-way ANOVA with Tukey’s HSD post hoc test (* *p* < 0.05, *** *p* < 0.001, **** *p* < 0.0001, ns = no significance).

Pathway activity scores representing major components of the gliovascular unit were compared across WHO grade II–IV gliomas (Figure 1). In the TCGA cohort, homeostatic astrocyte pathway activity was significantly lower in grade III than grade II (Tukey-adjusted *p* = 0.045), lower still in grade IV than grade III (*p* < 0.001), and the lowest overall comparing grade IV to grade II (*p* < 0.001). BM remodeling showed the same pattern of significant differences across all three pairwise comparisons (II vs. III, III vs. IV, and II vs. IV, all *p* < 0.001), as did endothelial angiogenesis. Reactive astrocytes, neuroinflammation, and ECM remodeling were each significantly higher in grade IV than in both lower grades (all *p* < 0.001). Astrocyte endfoot organization and BBB specialization did not differ significantly across grades. In the CGGA validation cohort, no pathway differed significantly between grade II and grade III (Figure S1). Significant differences were observed only for comparisons involving grade IV, specifically for reactive astrocytes (III vs. IV, *p* = 0.011; II vs. IV, *p* = 0.004), neuroinflammation (III vs. IV, *p* = 0.007; II vs. IV, *p* < 0.001), endothelial angiogenesis, ECM remodeling, and BM remodeling (all III vs. IV and II vs. IV, *p* < 0.001), and homeostatic astrocytes (II vs. IV only, *p* = 0.003). Grade associations for all eight pathways were concordant in direction and significance when recalculated using single-sample gene set enrichment analysis (ssGSEA) as an alternative scoring method, with two exceptions detailed in Table S1. Leave-one-gene-out analysis further demonstrated that the significant pathway-level grade associations were generally robust to individual gene removal, with directionality preserved across the significant pathways in both cohorts (Table S2).

To determine whether gliovascular pathway activity was also associated with molecular subtype, pathway activity scores were compared between IDH-mutant and IDH-wildtype tumors (Figure S2). In the TCGA cohort, reactive astrocyte (*p* < 0.001), neuroinflammation (*p* < 0.001), endothelial angiogenesis (*p* < 0.001), ECM remodeling (*p* < 0.001), BM remodeling (*p* < 0.001), and astrocyte endfoot organization (*p* < 0.001) pathway scores were significantly higher in IDH-wildtype tumors than in IDH-mutant tumors (Figure S2A). In contrast, homeostatic astrocyte and BBB specialization pathway scores did not differ significantly between IDH groups. Similar overall trends were observed in the CGGA validation cohort (Figure S2B). However, homeostatic astrocyte pathway activity was significantly reduced in IDH-wildtype tumors (*p* = 0.039), whereas astrocyte endfoot organization no longer differed significantly between molecular subtypes (*p* = 0.068) (Figure S2B).

### 3.2. Gene-Level Analysis Confirms Coordinated Pathway Remodeling

**Figure 2:**
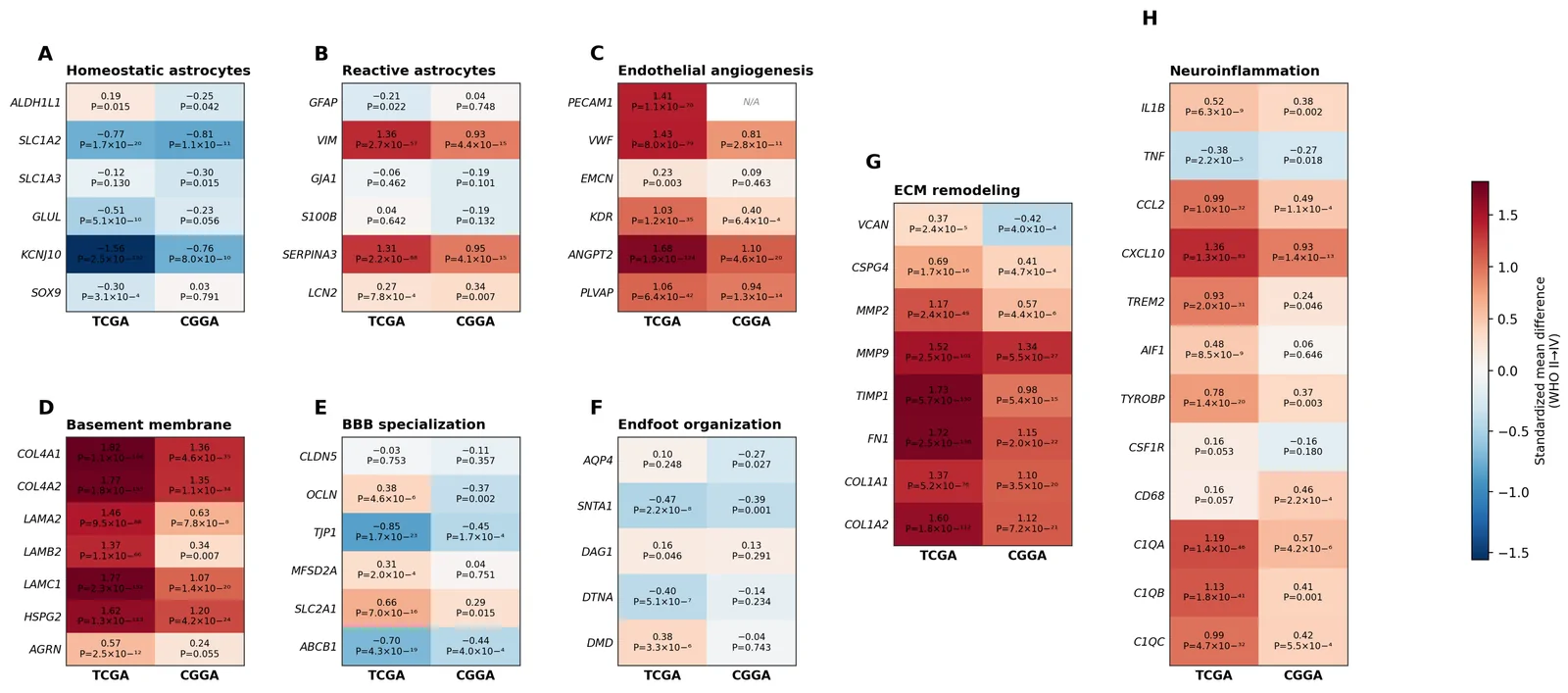
Gene-level validation of pathway activity scores, TCGA vs. CGGA. Standardized mean difference (WHO grade II vs. IV, z-scored expression) for each of 56 marker genes composing the eight pathway scores, grouped by pathway (**A**–**H**). Cell values show the standardized mean difference and *p*-value (Welch’s *t*-test) in TCGA (*n* = 800) and CGGA (*n* = 422). PECAM1 was not detected in the CGGA expression matrix (N/A).

To confirm that pathway-level associations were not driven disproportionately by a small subset of marker genes, standardized mean differences (WHO grade II versus WHO grade IV) were calculated for each of the 56 genes comprising the eight gliovascular pathway scores (Figure 2). The majority of genes demonstrated consistent directionality and similar effect sizes across both cohorts, supporting the robustness of the pathway-level analyses.

The BM remodeling pathway exhibited the greatest internal consistency, with all seven constituent genes demonstrating higher expression in grade IV than grade II tumors in both TCGA and CGGA (Figure 2D). The largest differences in mean standardized expression were observed for COL4A1 (1.82 in TCGA and 1.36 in CGGA), COL4A2 (1.77 and 1.35, respectively), and HSPG2 (1.62 and 1.20, respectively). Similarly, five of eight genes within the ECM remodeling pathway (FN1, MMP9, TIMP1, COL1A1, and COL1A2) exhibited consistent higher expression in grade IV tumors across both cohorts (Figure 2G). Within the homeostatic astrocyte pathway, KCNJ10 (−1.56 in TCGA and −0.76 in CGGA) and SLC1A2 (−0.77 and −0.81, respectively) demonstrated the largest reductions between grade II and grade IV, while VIM (1.36 and 0.93) and SERPINA3 (1.31 and 0.95) were the principal contributors to the reactive astrocyte pathway (Figure 2A,B). ANGPT2 (1.68 and 1.10) and CXCL10/CCL2 (1.36/0.99 in TCGA and 0.93/0.49 in CGGA, respectively) similarly represented the strongest contributors to the endothelial angiogenesis and neuroinflammation pathways (Figure 2C,H).

Within the astrocyte endfoot organization pathway, SNTA1 exhibited a consistent, significant reduction in grade IV compared with grade II tumors in both cohorts (difference in mean standardized expression = −0.47 in TCGA, *p* < 0.001; −0.39 in CGGA, *p* = 0.001). AQP4, the principal water channel mediating astrocyte-dependent glymphatic clearance, did not differ significantly between grade II and grade IV tumors in TCGA (difference = 0.10, *p* = 0.248) and showed only a small decrease in grade IV tumors in CGGA (difference = −0.27, *p* = 0.027) (Figure 2F).

### 3.3. Grade-Adjusted Pathway Interactions Identify a Conserved Gliovascular Remodeling Network

**Figure 3:**
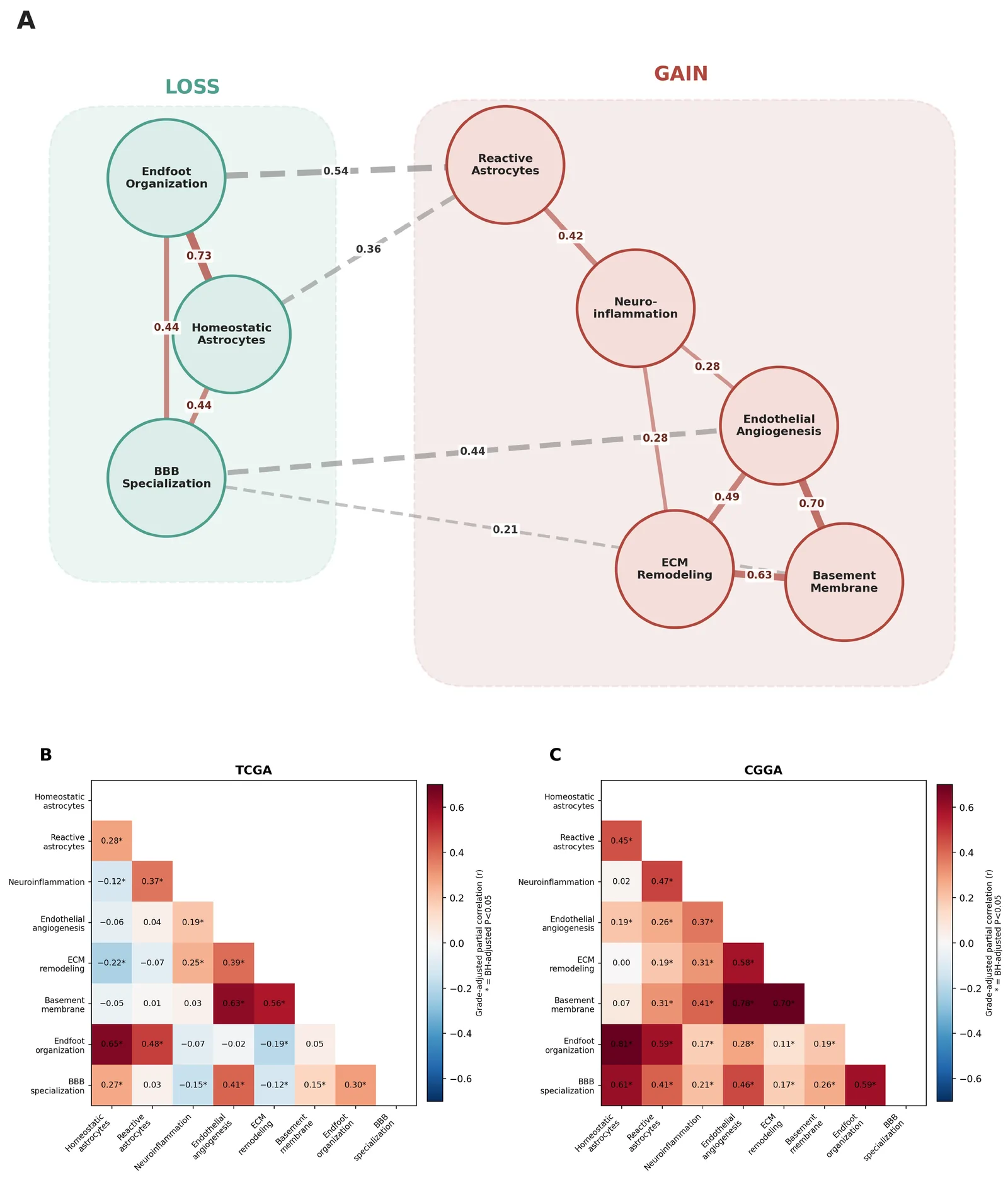
Grade-adjusted partial correlation structure among pathway scores. (**A**) Network diagram of pathway relationships that were BH-significant and replicated with the same sign in both TCGA and CGGA. Pathways separate into two coordinated programs, Loss (astrocyte-perivascular architecture) and Gain (reactive, inflammatory, and vascular remodeling), shown as shaded regions; edge width reflects mean correlation strength across cohorts. (**B**,**C**) Full pairwise grade-adjusted partial correlation matrices for all eight pathways in TCGA (**B**) and CGGA (**C**). Asterisks indicate BH-adjusted *p* < 0.05.

Comparison of grade-adjusted gliovascular pathway activity revealed a highly conserved network architecture across both cohorts (Figure 3). Rather than forming a single continuum of remodeling, the gliovascular unit segregated into two coordinated biological programs. The Loss program comprised homeostatic astrocytes, astrocyte endfoot organization, and BBB specialization, whereas the Gain program comprised reactive astrocytes, neuroinflammation, endothelial angiogenesis, ECM remodeling, and BM remodeling (Figure 3A). Correlations were consistently stronger within each program than between them (Figure 3B,C), supporting coordinated relationships among transcriptional programs associated with loss of gliovascular homeostasis and gain of inflammatory and vascular remodeling across glioma grades rather than uniform dysregulation of all pathways.

Within the Loss program, homeostatic astrocyte activity was strongly associated with astrocyte endfoot organization (r = 0.65 TCGA, 0.81 CGGA) and BBB specialization (r = 0.27, 0.61; all BH-adjusted *p* < 0.001) (Figure 3B,C). Within the Gain program, the strongest and most reproducible relationships were observed among ECM remodeling, BM remodeling, and endothelial angiogenesis (r = 0.39–0.78 across cohorts), identifying a tightly coordinated vascular remodeling core (Figure 3A–C). Neuroinflammation showed moderate positive associations with both reactive astrocytes (r = 0.37 TCGA, 0.47 CGGA) and the vascular remodeling pathways (r = 0.19–0.31), positioning it between astrocyte-associated and vascular-remodeling programs within the grade-adjusted correlation structure.

Although the Loss and Gain programs remained largely distinct, they were connected through two reproducible bridging relationships (Figure 3A). Reactive astrocytes demonstrated positive associations with both homeostatic astrocytes (r = 0.28 TCGA, 0.45 CGGA) and astrocyte endfoot organization (r = 0.48, 0.59), whereas BBB specialization remained positively associated with endothelial angiogenesis (r = 0.41, 0.46) and BM remodeling (r = 0.15, 0.26). These bridging relationships demonstrate that the Loss and Gain transcriptional programs remain interconnected despite their broader separation within the grade-adjusted network. Full pairwise Spearman correlations among all eight pathway scores, without grade adjustment, are provided in Table S6 and show a concordant pattern.

### 3.4. Transition-Specific Analysis Identifies Differential Remodeling Across Glioma Grades

**Figure 4:**
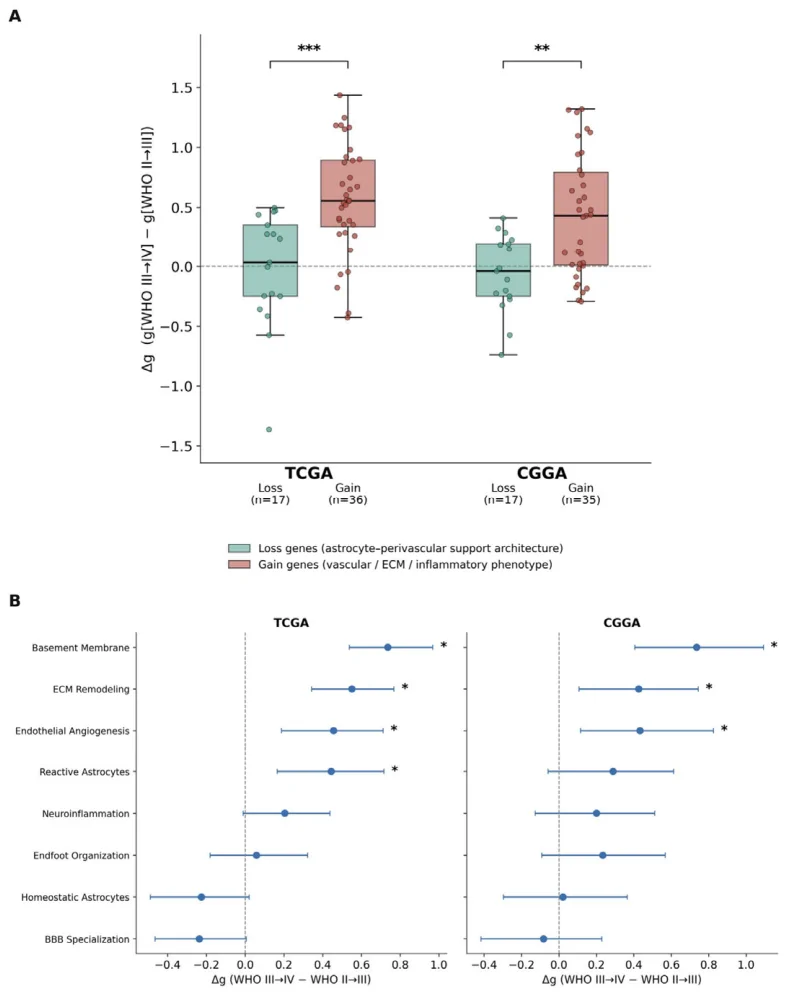
Grade-specific effect-size differences distinguish Loss and Gain gene programs. ((**A**) Per-gene difference in grade-specific effect size (Δg = Hedges’ g[WHO III–IV] − Hedges’ g[WHO II–III]) compared between genes classified a priori as reflecting loss of homeostatic architecture (*n* = 17) or gain of a reactive, inflammatory, and vascular phenotype (*n* = 36 TCGA, 35 CGGA). Mann–Whitney U test, TCGA *p* = 0.0001, CGGA *p* = 0.002. (**B**) Pathway-level Δg with bootstrap 95% CI for each of the eight pathways in TCGA and CGGA; asterisks indicate BH-adjusted *p* < 0.05.

Comparison of grade-specific effect sizes demonstrated that the magnitude of gliovascular differences varied between the WHO grade II–III and grade III–IV comparisons (Figure 4). Across both cohorts, genes associated with the Gain program exhibited significantly greater Δg values than genes associated with the Loss program (TCGA: *p* = 0.0001; CGGA: *p* = 0.002) (Figure 4A). These findings indicate that Gain-associated genes tended to show larger differences between grades III and IV relative to grades II and III than Loss-associated genes.

At the pathway level, BM remodeling, ECM remodeling, and endothelial angiogenesis showed positive Δg values in both cohorts, indicating larger effect sizes for the grade III–IV comparison than for the grade II–III comparison (Figure 4B). BM remodeling showed the strongest and most statistically robust difference, remaining significant after multiple-testing correction in both TCGA (Δg = 0.74, BH-adjusted *p* = 0.002) and CGGA (Δg = 0.74, BH-adjusted *p* = 0.004). In TCGA, ECM remodeling (Δg = 0.55, BH-adjusted *p* = 0.002), endothelial angiogenesis (Δg = 0.46, BH-adjusted *p* = 0.005), and reactive astrocytes (Δg = 0.44, BH-adjusted *p* = 0.016) also remained significant after correction. In CGGA, ECM remodeling and endothelial angiogenesis showed similar positive Δg values but did not remain significant after multiple-testing correction (both BH-adjusted *p* = 0.085). The remaining pathways did not demonstrate significant differences in effect size between the two grade comparisons.

### 3.5. Gliovascular Signatures Covary with Neftel-Derived Transcriptional Programs

To examine whether gliovascular-associated transcriptional programs covaried with expression patterns resembling the GBM cell states described by Neftel et al., pathway scores were compared with bulk Neftel-derived transcriptional scores in TCGA and CGGA (Figure S3). Homeostatic astrocyte and astrocyte endfoot-organization signatures showed the strongest positive associations with the AC-like program, whereas ECM-remodeling and several inflammatory or vascular-remodeling signatures were positively associated with the MES-like program.

We next assessed whether these relationships were driven by shared gene membership. Eleven genes overlapped between the gliovascular signatures and the full Neftel meta-modules, affecting five of the eight gliovascular signatures. After removal of overlapping genes, the homeostatic astrocyte signature remained strongly associated with the AC-like program in TCGA (r = 0.72, BH-adjusted *p* < 0.001) and CGGA (r = 0.55, BH-adjusted *p* < 0.001). The endfoot-organization signature likewise remained positively associated with AC-like transcription in TCGA (r = 0.61, BH-adjusted *p* < 0.001) and CGGA (r = 0.39, BH-adjusted *p* < 0.001). The ECM-remodeling signature remained strongly associated with the MES-like program in both TCGA (r = 0.62, BH-adjusted *p* < 0.001) and CGGA (r = 0.65, BH-adjusted *p* < 0.001). In contrast, removal of overlapping genes reduced the reactive astrocyte-associated signature to a single non-overlapping gene, LCN2, and its association with the MES-like program was substantially attenuated. Accordingly, the reactive astrocyte–MES-like relationship was considered sensitive to shared gene content and is interpreted cautiously. Together, these analyses support reproducible covariance between selected gliovascular signatures and Neftel-derived transcriptional programs that persists after accounting for shared gene membership (Table S3).

### 3.6. Representative Gliovascular Genes Demonstrate Distinct Spatial Organization Within Human GBM

**Figure 5:**
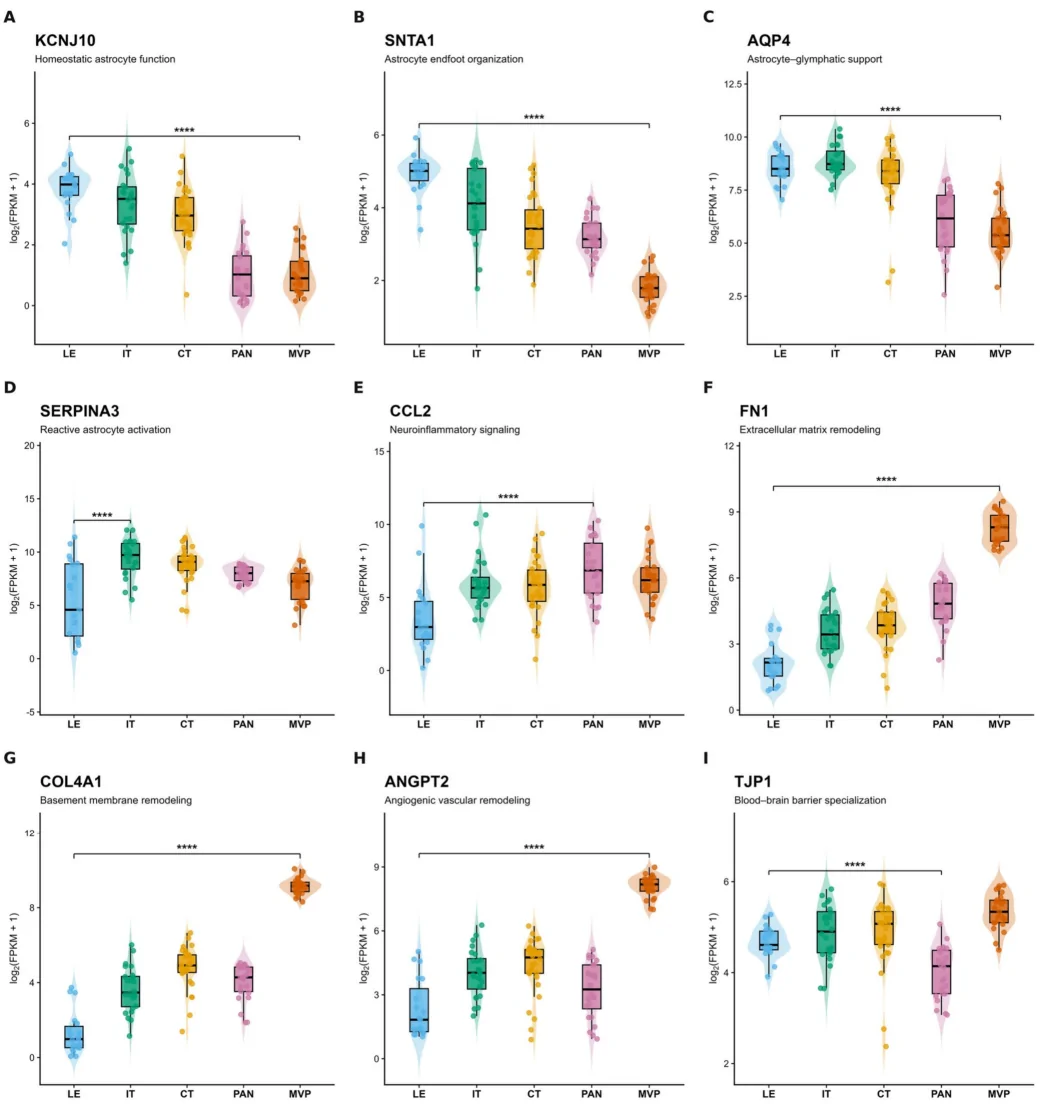
Spatial distribution of representative gliovascular genes across anatomically defined GBM regions. Regional expression of nine representative genes (**A**–**I**), one to two per pathway, evaluated across the leading edge (LE), infiltrating tumor (IT), cellular tumor (CT), pseudopalisading cells surrounding necrosis (PAN), and microvascular proliferation (MVP) using the Ivy Glioblastoma Atlas Project. Expression values are log_2_(FPKM + 1). Regional differences were assessed using linear mixed-effects models (region as fixed effect, tumor donor as random intercept) followed by Tukey-adjusted pairwise comparisons; brackets denote a single selected, biologically informative regional contrast per gene, not the full comparison matrix, **** = *p* < 0.0001 (full pairwise results in Table S5).

We next examined the regional organization of representative gliovascular genes across anatomically defined GBM compartments (Figure 5). Genes associated with homeostatic astrocyte function and astrocyte endfoot organization were preferentially enriched at the tumor margin (Figure 5A–C). KCNJ10, a key regulator of astrocytic potassium buffering, was most highly expressed at the LE and showed lower expression across CT, PAN, and MVP, with significantly lower expression in PAN and MVP than in LE, IT, and CT (all *p* < 0.001). Similarly, SNTA1 showed lower expression in more centrally located tumor regions, with the lowest expression in MVP and significant differences across nearly all regional comparisons. In contrast, AQP4 remained relatively preserved throughout LE, IT, and CT and was lower within PAN and MVP, indicating a more heterogeneous regional distribution than either KCNJ10 or SNTA1.

Reactive astrocyte- and inflammatory-associated gene expression was enriched within infiltrating and perinecrotic tumor regions (Figure 5D,E). SERPINA3 showed minimal expression at the LE but increased markedly within IT, reaching maximal expression in IT, whereas CCL2 exhibited peak expression within PAN adjacent to necrosis. Both markers showed significantly greater expression in selected infiltrating and perinecrotic regions than at the tumor margin, supporting regional enrichment of inflammatory-associated transcription within the GBM microenvironment.

Genes associated with vascular remodeling exhibited the strongest regional differences (Figure 5F–I). FN1 was most highly expressed within MVP, while COL4A1 and ANGPT2 demonstrated similar regional distributions, with both showing their highest expression in MVP. In contrast, the BBB specialization marker TJP1 showed selective reduction within PAN while remaining relatively preserved in LE, IT, CT, and MVP. Complete mixed-effects-model results and Tukey-adjusted pairwise regional comparisons are provided in Tables S4 and S5, respectively.

### 3.7. Gliovascular Remodeling Pathways Do Not Provide Robust Independent Prognostic Information

**Figure 6:**
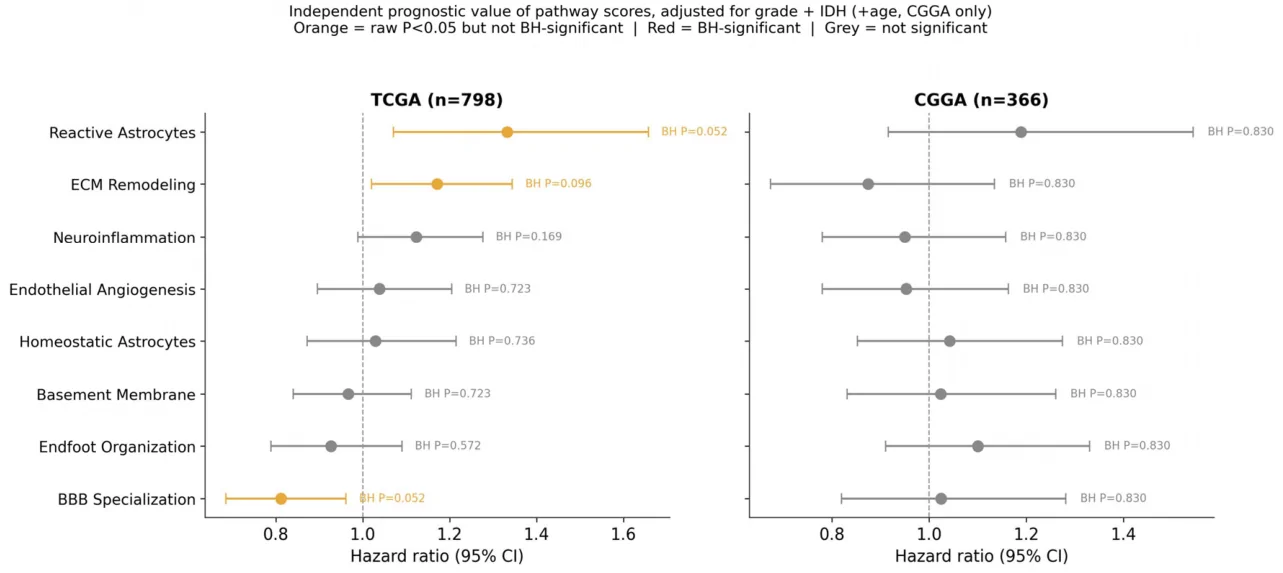
Independent prognostic value of pathway scores. Forest plot of hazard ratios (95% CI) for overall survival for each of the eight pathway scores, from Cox proportional hazards models adjusted for WHO grade and IDH status (TCGA, *n* = 798; CGGA, *n* = 366, additionally adjusted for age). Orange, nominal *p* < 0.05 but not BH-significant; grey, not significant. *p*-values BH-adjusted within cohort across the eight pathways.

The prognostic significance of each gliovascular unit pathway was also evaluated (Figure 6). In the TCGA cohort, no pathway remained significantly associated with overall survival after multiple-testing correction. Reactive astrocyte pathway activity (HR = 1.33, 95% CI 1.07–1.66; BH-adjusted *p* = 0.052) and BBB specialization pathway activity (HR = 0.81, 95% CI 0.69–0.96; BH-adjusted *p* = 0.052) approached, but did not reach, the adjusted significance threshold. ECM remodeling showed a nominal association with poorer survival before multiple-testing correction (HR = 1.17, 95% CI 1.02–1.34; *p* = 0.036), but this association was not significant after correction. Homeostatic astrocytes, astrocyte endfoot organization, neuroinflammation, BM remodeling, and endothelial angiogenesis were not independently associated with overall survival following multivariable adjustment.

In the independent CGGA cohort, none of the eight gliovascular pathways demonstrated a significant association with overall survival after adjustment for age, tumor grade, and IDH mutation status. Proportional-hazards testing identified significant global non-proportionality in both cohorts, driven predominantly by grade and IDH status. Pathway-specific tests showed no evidence of non-proportionality for any pathway in CGGA and for six of eight pathways in TCGA, with BM and ECM remodeling showing evidence of non-proportionality in TCGA. Accordingly, the survival findings were interpreted cautiously. Collectively, these findings indicate that gliovascular pathway activity did not provide robust prognostic information beyond established clinical and molecular variables.

### 3.8. An Integrative Model Summarizes Coordinated Gliovascular Remodeling Across Glioma Grades

**Figure 7:**
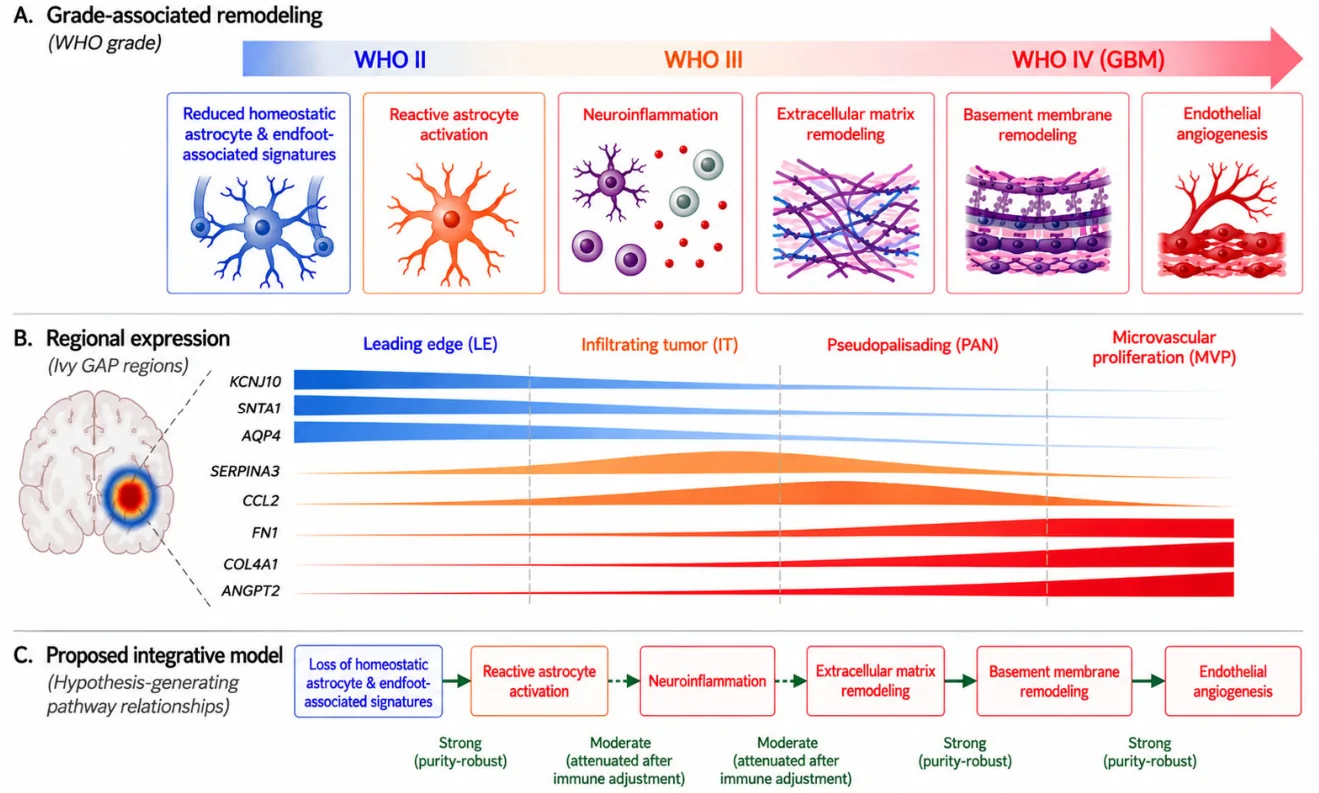
Proposed integrated model of gliovascular remodeling across glioma grades. (**A**) Grade-associated remodeling pattern across WHO grades, illustrating the six core pathways and their representative marker genes. (**B**) Regional expression patterns of representative genes across Ivy GAP anatomical regions (LE→IT→PAN→MVP). (**C**) Integrative remodeling model summarizing pathway relationships, with edge labels indicating the strength of statistical support: strong (persists after tumor-purity adjustment) and moderate (attenuated after immune-infiltration adjustment). Arrows in Panel C represent proposed relationships and do not establish temporal ordering or causal direction.

The principal findings of this study were integrated into a hypothesis-generating model summarizing the grade-associated, regional, and pathway-level relationships observed across the analyses (Figure 7). The proposed model organizes these findings from homeostatic astrocyte-associated signatures through reactive, inflammatory, matrix, and vascular-remodeling programs; this ordering represents an integrative interpretation of the cross-sectional data rather than an observed temporal sequence. Homeostatic astrocyte activity was linked to reactive astrocyte activation (r = 0.28 TCGA, 0.45 CGGA), a relationship that remained stable after adjustment for tumor purity, supporting an association between these transcriptional programs that was not attributable solely to differences in estimated tumor purity.

Reactive astrocyte activation was linked to neuroinflammation (r = 0.37, 0.47), and neuroinflammation was also associated with ECM remodeling (r = 0.25, 0.31); both relationships, however, were substantially attenuated (79–97%) after adjustment for general immune infiltration, indicating that these connections are strongly influenced by shared immune cell content and do not establish specific regulatory relationships among astrocyte reactivity, neuroinflammation, and matrix remodeling. A negative association between homeostatic astrocytes and neuroinflammation (r = −0.12 TCGA) did not replicate in CGGA (r = 0.02) and was therefore not included in the model, though it persisted after immune adjustment in TCGA (r = −0.19) and may warrant reconsideration in future work.

The vascular-remodeling component of the model was the most robustly supported. ECM remodeling was strongly linked to BM remodeling (r = 0.56, 0.70), which was strongly associated with endothelial angiogenesis (r = 0.63, 0.78); both relationships remained stable after adjustment for tumor purity. Together, these observations support a proposed framework in which homeostatic astrocyte-associated, inflammatory, and vascular-remodeling transcriptional programs are interconnected across glioma grades, with the strength of statistical support varying across components of the model. The cross-sectional design does not establish the temporal ordering or causal direction of these relationships.

## 4. Discussion

This study integrates curated gliovascular-associated transcriptional programs across independent diffuse glioma cohorts and anatomically annotated GBM regions to characterize coordinated gliovascular remodeling across disease grades. Although disruption of astrocytes, BBB integrity, angiogenesis, ECM remodeling, and neuroinflammation have each been independently described in glioma, our findings suggest that these alterations are organized into a reproducible biological remodeling program rather than representing isolated pathological processes [11,12,18,19,20,28]. Importantly, this model extends beyond the traditional concept of BBB disruption. While BBB dysfunction primarily reflects increased vascular permeability resulting from endothelial barrier breakdown [28], the gliovascular unit encompasses the broader astrocytic, vascular, BM, and ECM architecture that supports neurovascular homeostasis and provides the structural framework for perivascular glymphatic transport [1,3,4].

Gliovascular remodeling across glioma grades was coordinated across this broader structural and functional unit rather than occurring as a series of independent alterations. Genes reflecting loss of homeostatic astrocyte architecture and genes reflecting gain of a reactive, inflammatory, and vascular remodeling phenotype showed significantly different grade-specific effect-size patterns in both cohorts. At the pathway level, BM remodeling showed the most robust evidence of a larger effect between grades III and IV than between grades II and III, while ECM remodeling and endothelial angiogenesis showed similar positive Δg patterns across both cohorts. Regional Ivy GAP analyses further demonstrated that this remodeling program is anatomically compartmentalized within the GBM TME, with homeostatic astrocyte markers concentrated at the tumor margin and vascular/matrix remodeling genes concentrated within regions of MVP. Rather than representing independent pathological events, these alterations appear to reflect coordinated reorganization of a single biological system responsible for maintaining normal gliovascular homeostasis.

Among the pathways examined, homeostatic astrocyte identity showed a consistent, grade-associated decline in both cohorts, and genes reflecting loss of homeostatic architecture showed smaller grade III–IV relative to grade II–III effect-size differences than genes reflecting gain of a reactive, inflammatory, and vascular phenotype. This pattern indicates that loss of homeostatic astrocyte-associated transcription is evident across glioma grades, whereas Gain-associated programs show comparatively larger differences at higher grades. Among these genes, KCNJ10 and SNTA1 demonstrated the most reproducible decline across independent cohorts, whereas AQP4 expression remained comparatively stable. This distinction is biologically important because glymphatic function depends not only on AQP4 abundance, but also on its polarized localization to perivascular astrocyte endfeet through the dystrophin-associated protein complex, of which SNTA1 is a critical component [29,30,31]. Experimental studies have demonstrated that loss of perivascular AQP4 polarization can impair glymphatic exchange despite preservation of overall AQP4 expression [29,32]. These findings suggest that grade-associated disruption of gliovascular homeostasis may involve altered astrocyte organization rather than loss of astrocytic identity alone.

Although glymphatic function was not directly measured in the present study, impaired glymphatic transport has previously been demonstrated in GBM and has been associated with increasing edema, neuroinflammation, and disruption of perivascular fluid movement [6,7]. The present findings provide a potential molecular framework for these observations by linking disruption of astrocyte-associated transcriptional programs with inflammatory and vascular remodeling across glioma grades. Future experimental studies will be required to determine whether disruption of astrocyte endfoot organization contributes directly to impaired glymphatic clearance and whether preservation of this architecture could mitigate remodeling of the TME.

In the present study, higher-grade gliomas showed a transcriptional landscape characterized by reduced astrocyte-homeostatic signatures alongside increasingly prominent vascular-remodeling signatures. The larger grade III–IV effect sizes observed for ECM remodeling, BM remodeling, and angiogenic programs are consistent with the established pathology of advanced GBM, in which hypoxia, necrosis, and metabolic stress promote aberrant angiogenesis and profound remodeling of the TME [33,34]. Regional Ivy GAP analyses provided complementary anatomical context, demonstrating that genes associated with ECM remodeling (FN1), BM remodeling (COL4A1), and angiogenesis (ANGPT2) were preferentially enriched within regions of MVP, whereas homeostatic astrocyte markers predominated at the LE and IT. This regional organization parallels the grade-associated pattern identified in the bulk transcriptomic analyses, with astrocyte-associated signatures predominating at the tumor margin and vascular-remodeling signatures enriched within MVP. Together, these findings are consistent with structural reorganization of the gliovascular unit across glioma grades and anatomical regions. Consistent with the reproducible association between the ECM-remodeling signature and the Neftel-derived MES-like transcriptional program, these findings suggest that vascular and extracellular-matrix remodeling covary with broader mesenchymal-associated transcription in advanced glioma [35,36].

Several limitations should be considered when interpreting these findings. First, all transcriptomic analyses were performed using bulk RNA sequencing and therefore cannot distinguish transcriptional changes occurring within individual cell populations from alterations in cellular composition during glioma grade. This limitation is particularly relevant for genes such as AQP4, whose biological function depends on polarized localization within astrocyte endfeet rather than transcript abundance alone [32]. Consequently, the relatively stable AQP4 expression observed in this study should not be interpreted as preservation of normal glymphatic function. Second, the TCGA and CGGA cohorts span different WHO classification eras, and the molecular information required to retrospectively harmonize all cases according to the 2021 WHO integrated classification was not uniformly available. Tumors were therefore analyzed according to their original grade and available molecular annotations, and some cases may be classified differently under current diagnostic criteria. Finally, the proportional-hazards assumption was not satisfied globally in the survival models in either cohort, driven predominantly by non-proportional effects of grade and IDH status. Although most pathway terms did not individually show evidence of non-proportionality, the survival findings should therefore be interpreted cautiously.

The Ivy GAP analyses and tumor-purity adjustments provided complementary support for the observed gliovascular associations; however, direct measurements of glymphatic transport, cerebrospinal fluid dynamics, and protein localization were beyond the scope of the present study. These findings identify coordinated transcriptional associations across glioma grades and anatomical regions but do not establish temporal ordering or causal relationships among astrocyte dysfunction, inflammation, vascular remodeling, and impaired glymphatic function. Experimental glioma models incorporating functional imaging and mechanistic manipulation of the gliovascular unit will be necessary to determine how these processes interact and whether they contribute directly to disease progression. The Neftel-derived analyses carry an additional interpretive limitation. The Neftel framework was developed primarily from GBM, whereas the TCGA and CGGA cohorts analyzed here include diffuse gliomas across multiple grades and molecular backgrounds. Accordingly, Neftel-derived scores were used as measures of bulk transcriptional resemblance to AC-like, OPC-like, NPC-like, MES-like, and cycling programs rather than as direct estimates of malignant-cell state composition. Gene-set overlap also influenced selected associations; although the major homeostatic astrocyte–AC-like, endfoot–AC-like, and ECM-remodeling–MES-like relationships persisted after removal of overlapping genes, the reactive astrocyte–MES-like relationship was substantially attenuated and should therefore be interpreted cautiously.

An important implication of this molecular framework is the potential intersection between gliovascular remodeling, glymphatic function, and sleep physiology. Although the glymphatic system is increasingly recognized as a critical regulator of brain waste clearance and neuroimmune homeostasis, relatively little is known about its relationship with glioma biology. Glymphatic transport is most active during sleep, and the high prevalence of sleep disturbance among patients with GBM raises the possibility of a bidirectional relationship between sleep disruption and tumor-associated gliovascular dysfunction [36,37,38,39,40]. Tumor-associated alterations in the vascular, inflammatory, and perivascular environment may contribute to disruption of normal sleep physiology, while impaired sleep could in turn influence glymphatic transport, neuroinflammatory signaling, and vascular homeostasis. The current analyses cannot establish the direction or causality of these relationships because sleep and glymphatic function were not directly measured. Nevertheless, the grade-associated remodeling framework presented here provides a biological foundation for investigating how sleep disruption, glymphatic dysfunction, and gliovascular remodeling may interact in glioma. By defining the molecular organization of the gliovascular unit across glioma grades and anatomical regions, this work establishes a foundation for connecting tumor biology with sleep physiology through the shared biology of the glymphatic system.

## 5. Conclusions

Diffuse glioma is characterized by coordinated, grade-associated remodeling of the gliovascular unit rather than isolated disruption of individual biological pathways. By defining the grade-associated and regional organization of transcriptional signatures associated with astrocyte dysfunction, inflammatory activation, and vascular remodeling, this study provides a systems-level framework for understanding glioma biology beyond conventional models of BBB dysfunction. These findings are limited by the retrospective, transcriptomic nature of the study and should not be interpreted as demonstrating longitudinal progression, temporal ordering, or causal changes in gliovascular function. This framework establishes a foundation for future studies investigating how gliovascular remodeling, glymphatic dysfunction, and sleep disturbance interact to influence GBM progression and therapeutic response.

## Figures and Tables

**Table 1:** Biological functions of curated marker genes representing components of the gliovascular unit.

Pathway	Genes	Rationale/Shared Function in the Gliovascular Unit
Astrocyte endfoot organization	*AQP4*	Principal astrocytic water channel mediating perivascular water movement and CSF–interstitial fluid exchange.
	*SNTA1*, *DAG1*, *DTNA*, *DMD*	Components of the dystrophin-associated protein complex that anchor and stabilize polarized AQP4 at astrocyte endfeet and link endfeet to the vascular basement membrane.
Homeostatic astrocytes	*ALDH1L1*, *SOX9*	Markers of mature, homeostatic astrocyte identity.
	*SLC1A2*, *SLC1A3*, *GLUL*	Glutamate transport and recycling machinery supporting extracellular glutamate clearance and neuronal homeostasis.
	*KCNJ10*	Astrocytic potassium channel required for extracellular potassium buffering and normal perivascular ionic-water balance.
Reactive astrocytes	*GFAP*, *VIM*	Intermediate filament and cytoskeletal proteins upregulated with astrocyte activation and reactive gliosis.
	*GJA1*, *S100B*	Gap-junction and calcium-binding proteins associated with altered astrocyte network coupling and activation-associated signaling.
	*SERPINA3*, *LCN2*	Secreted acute-phase and inflammatory mediators associated with chronic reactive astrocyte states.
BBB specialization	*CLDN5*, *OCLN*, *TJP1*	Tight-junction complex components that restrict paracellular permeability and maintain junctional organization at the BBB.
	*MFSD2A*, *SLC2A1*	Specialized endothelial transporters that suppress transcytosis and support nutrient transport across brain endothelium.
	*ABCB1*	P-glycoprotein efflux transporter limiting entry and promoting removal of xenobiotics across the BBB.
Endothelial angiogenesis	*PECAM1*, *VWF*, *EMCN*	Endothelial adhesion and glycocalyx proteins marking vascular endothelial abundance and identity.
	*KDR*, *ANGPT2*	VEGF-pathway signaling mediators driving endothelial proliferation, vessel destabilization, and angiogenic sprouting.
	*PLVAP*	Endothelial permeability-associated protein marking fenestrated or pathologically permeable vasculature.
Basement membrane	*COL4A1*, *COL4A2*	Type IV collagen chains providing structural support to the vascular basement membrane.
	*LAMA2*, *LAMB2*, *LAMC1*	Laminin chains required for laminin assembly, basement membrane organization, and astrocyte endfoot attachment.
	*HSPG2*, *AGRN*	Basement membrane proteoglycans that provide structural support and maintain astrocyte endfoot attachment and perivascular AQP4 polarization.
ECM remodeling	*VCAN*, *CSPG4*	Chondroitin sulfate proteoglycans contributing to ECM expansion, organization, and the perivascular niche.
	*MMP2*, *MMP9*, *TIMP1*	Matrix metalloproteinases and their endogenous inhibitor regulating ECM degradation and turnover.
	*FN1*, *COL1A1*, *COL1A2*	Fibrillar matrix proteins associated with matrix deposition, fibrosis, and increased ECM stiffness.
Neuroinflammation	*IL1B*, *TNF*	Pro-inflammatory cytokines that activate glial inflammatory responses and can impair blood–brain barrier function.
	*CCL2*, *CXCL10*	Chemokines that recruit and activate monocytes, macrophages, and immune cells within the tumor and perivascular microenvironment.
	*TREM2*, *TYROBP*, *CSF1R*	Innate immune receptors and adaptor signaling controlling microglial and macrophage survival, proliferation, and activation.
	*AIF1*, *CD68*	Markers of activated, phagocytic microglia and macrophages.
	*C1QA*, *C1QB*, *C1QC*	Complement components associated with activated microglia and innate immune signaling.

## Data Availability

Publicly available datasets were analyzed in this study. TCGA transcriptomic and clinical data are available through the National Cancer Institute Genomic Data Commons (GDC) Data Portal: https://portal.gdc.cancer.gov (accessed on 1 July 2026). CGGA transcriptomic and clinical data are available through the Chinese Glioma Genome Atlas: http://www.cgga.org.cn (accessed on 1 July 2026). Regional transcriptomic data from the Ivy Glioblastoma Atlas Project (Ivy GAP) are available through the Allen Institute for Brain Science: https://glioblastoma.alleninstitute.org (accessed on 17 July 2026). Processed analytical data, curated pathway definitions, analysis code, and supporting results are publicly available through the project GitHub repository: https://github.com/JAllgood98/Gliovascular-glioma-transcriptomics.

## Supplementary Materials

The following supporting information can be downloaded at: https://www.mdpi.com/article/10.3390/cancers18182908/s1, Figure S1. Pathway activity scores across WHO glioma grade, CGGA validation cohort. Violin plots of eight pathway activity scores (A–H: homeostatic astrocytes, reactive astrocytes, neuroinflammation, endothelial angiogenesis, ECM remodeling, basement membrane, endfoot organization, BBB specialization) stratified by WHO grade (II, III, IV) in the independent CGGA validation cohort (*n* = 422). Box plots show median and interquartile range; individual points show single samples. Significance was assessed using one-way ANOVA followed by Tukey’s honestly significant difference (HSD) post-hoc test for pairwise comparisons between grades; only statistically significant pairwise comparisons are displayed (* *p* < 0.05, ** *p* < 0.01, *** *p* < 0.001, **** *p* < 0.0001); Figure S2. Validation of IDH-associated pathway scores, TCGA and CGGA. (A) TCGA and (B) CGGA: violin plots of the eight pathway scores by IDH mutation status (mutant vs. wildtype). Significance from Welch’s *t*-test (* *p* < 0.05, *** *p* < 0.001, **** *p* < 0.0001; ns, not significant); Figure S3. Pathway associations with Neftel-derived transcriptional programs. Bubble plot of Pearson correlations between the eight pathway scores and five malignant cell-state scores (AC-like, OPC-like, NPC-like, MES-like, cycling) described by Neftel et al., in TCGA and CGGA. Circle size indicates |r|; color indicates direction and magnitude; Figure S4. Per-gene grade-specific effect-size differences by Loss/Gain category. (A) TCGA and (B) CGGA: per-gene difference in grade-specific effect size (Δg = Hedges’ g[WHO III–IV] − Hedges’ g[WHO II–III], bootstrap 95% CI) for all 53 core-category genes (Loss, teal; Gain, red), individually. Asterisks indicate BH-adjusted *p* < 0.05 for that gene’s Δg; Figure S5. Cell-type expression specificity of the 55 gliovascular marker genes, single-cell GBM reference atlas (338,564 cells). Dot plot showing the percentage of cells expressing each marker gene (dot size) and relative, row-scaled expression level (color) across annotated cell types, including malignant cell states (AC-like, MES-like, NPC-like, OPC-like), non-malignant glia (astrocyte, oligodendrocyte, oligodendrocyte precursor cell [OPC], radial glia [RG]), vascular cells (endothelial, mural), myeloid populations (microglia-derived and monocyte-derived tumor-associated macrophages [TAM-MG, TAM-BDM], monocytes [Mono], dendritic cells [DC]), lymphoid populations (CD4/CD8 T cells, natural killer [NK] cells, B cells, plasma B cells, mast cells), and neurons. Genes are grouped by their assigned pathway (Table 1); Table S1. Sensitivity of grade-associated gliovascular pathway effects to alternative ssGSEA scoring; Table S2. Leave-one-gene-out sensitivity analysis of grade-associated gliovascular pathway scores; Table S3. Sensitivity analysis of associations between gliovascular signatures and Neftel-derived transcriptional programs after removal of overlapping genes; Table S4. Mixed-effects model results for regional expression of representative gliovascular genes in Ivy GAP; Table S5. Pairwise regional comparisons for representative gliovascular genes, Ivy GAP; Table S6. Pairwise Spearman correlations among gliovascular pathway scores; Table S7. Robustness of core-chain partial correlations to tumor-purity adjustment, TCGA.

## Abbreviations

The following abbreviations are used in this manuscript:

AC-like	Astrocyte-like
BBB	Blood–brain barrier
BM	Basement membrane
CGGA	Chinese Glioma Genome Atlas
CT	Cellular tumor
ECM	Extracellular matrix
GBM	Glioblastoma
HR	Hazard ratio
IDH	Isocitrate dehydrogenase
IT	Infiltrating tumor
LE	Leading edge
MES-like	Mesenchymal-like
MVP	Microvascular proliferation
NPC-like	Neural progenitor cell-like
OPC-like	Oligodendrocyte progenitor cell-like
PAN	Pseudopalisading cells around necrosis
TCGA	The Cancer Genome Atlas
TME	Tumor microenvironment
WHO	World Health Organization

## References

[1] Jessen N.A., Munk A.S.F., Lundgaard I., Nedergaard M. (2015). The glymphatic system: A beginner’s guide. Neurochem. Res..

[2] Hablitz L.M., Nedergaard M. (2021). The Glymphatic system: A novel component of fundamental neurobiology. J. Neurosci..

[3] Abbott N.J., Pizzo M.E., Preston J.E., Janigro D., Thorne R.G. (2018). The role of brain barriers in fluid movement in the CNS: Is there a ‘glymphatic’ system?. Acta Neuropathol..

[4] Zlokovic B.V. (2011). Neurovascular pathways to neurodegeneration in Alzheimer’s disease and other disorders. Nat. Rev. Neurosci..

[5] Sweeney M.D., Ayyadurai S., Zlokovic B.V. (2016). Pericytes of the neurovascular unit: Key functions and signaling pathways. Nat. Neurosci..

[6] Jia L., Chen Y., Li H., Zhao K., Ge S., Wang C., Zhao J., Li F., Zhang L., Yao A. (2025). The glymphatic system in neurodegenerative diseases and brain tumors: Mechanistic insights, biomarker advances, and therapeutic opportunities. Acta Neuropathol. Commun..

[7] Kaur J., Ding G., Zhang L., Lu Y., Luo H., Li L., Boyd E., Li Q., Wei M., Zhang Z. (2023). Imaging glymphatic response to glioblastoma. Cancer Imaging.

[8] Gao M., Liu Z., Zang H., Wu X., Yan Y., Lin H., Yuan J., Liu T., Zhou Y., Liu J. (2024). A histopathologic correlation study evaluating glymphatic function in brain tumors by multiparametric MRI. Clin. Cancer Res..

[9] Fiallo Arroyo J., Leon-Rojas J.E. (2026). The glymphatic–immune axis in glioblastoma: Mechanistic insights and translational opportunities. Int. J. Mol. Sci..

[10] Nduom E.K., Yang C., Merrill M.J., Zhuang Z., Lonser R.R. (2013). Characterization of the blood-brain barrier of metastatic and primary malignant neoplasms. J. Neurosurg..

[11] Lee J., Lund-Smith C., Borboa A., Gonzalez A.M., Baird A., Eliceiri B.P. (2009). Glioma-induced remodeling of the neurovascular unit. Brain Res..

[12] Henrik Heiland D., Ravi V.M., Behringer S.P., Frenking J.H., Wurm J., Joseph K., Garrelfs N.W., Strähle J., Heynckes S., Grauvogel J. (2019). Tumor-associated reactive astrocytes aid the evolution of immunosuppressive environment in glioblastoma. Nat. Commun..

[13] van den Bent M.J., French P.J., Brat D., Tonn J.C., Touat M., Ellingson B.M., Young R.J., Pallud J., von Deimling A., Sahm F. (2024). The biological significance of tumor grade, age, enhancement, and extent of resection in IDH-mutant gliomas: How should they inform treatment decisions in the era of IDH inhibitors?. Neuro-Oncology.

[14] Reuss D.E. (2023). Updates on the WHO diagnosis of IDH-mutant glioma. J. Neuro-Oncol..

[15] Fang K., Wang C., Zhang Y., Wu S., Liu L., Wen X. (2025). Noninvasive assessment of glymphatic dysfunction and IDH mutation in glioma with DTI-ALPS and DTI metrics. Quant. Imaging Med. Surg..

[16] Zhang C.-b., Wang Z.-l., Liu H.-j., Wang Z., Jia W. (2023). Characterization of tumor-associated reactive astrocytes in gliomas by single-cell and bulk tumor sequencing. Front. Neurol..

[17] Wei Y., Chen D., Zhang Q., You F., Fu Y., Zheng L., Zhang L., Zhang N., Liang G., Yang J. (2025). ECM-based molecular subtypes define prognostic, EMT status, and therapeutic diversity in IDH-mutant gliomas. npj Precis. Oncol..

[18] Cui X., Huo D., Wang Q., Wang Y., Liu X., Zhao K., You Y., Zhang J., Kang C. (2024). RUNX1/NPM1/H3K4me3 complex contributes to extracellular matrix remodeling via enhancing FOSL2 transcriptional activation in glioblastoma. Cell Death Dis..

[19] Chen Z., Feng X., Herting C.J., Garcia V.A., Nie K., Pong W.W., Rasmussen R., Dwivedi B., Seby S., Wolf S.A. (2017). Cellular and molecular identity of tumor-associated macrophages in glioblastoma. Cancer Res..

[20] Sa J.K., Chang N., Lee H.W., Cho H.J., Ceccarelli M., Cerulo L., Yin J., Kim S.S., Caruso F.P., Lee M. (2020). Transcriptional regulatory networks of tumor-associated macrophages that drive malignancy in mesenchymal glioblastoma. Genome Biol..

[21] Ceccarelli M., Barthel F.P., Malta T.M., Sabedot T.S., Salama S.R., Murray B.A., Morozova O., Newton Y., Radenbaugh A., Pagnotta S.M. (2016). Molecular profiling reveals biologically discrete subsets and pathways of progression in diffuse glioma. Cell.

[22] Zhao Z., Zhang K.-N., Wang Q., Li G., Zeng F., Zhang Y., Wu F., Chai R., Wang Z., Zhang C. (2021). Chinese Glioma Genome Atlas (CGGA): A comprehensive resource with functional genomic data from Chinese glioma patients. Genom. Proteom. Bioinform..

[23] Lattke M., Guillemot F. (2022). Understanding astrocyte differentiation: Clinical relevance, technical challenges, and new opportunities in the omics era. WIREs Mech. Dis..

[24] Olsen M.L., Sontheimer H. (2008). Functional implications for Kir4.1 channels in glial biology: From K^+^ buffering to cell differentiation. J. Neurochem..

[25] Thomsen M.S., Routhe L.J., Moos T. (2017). The vascular basement membrane in the healthy and pathological brain. J. Cereb. Blood Flow Metab..

[26] Daneman R., Prat A. (2015). The blood–brain barrier. Cold Spring Harb. Perspect. Biol..

[27] Neftel C., Laffy J., Filbin M.G., Hara T., Shore M.E., Rahme G.J., Richman A.R., Silverbush D., Shaw M.L., Hebert C.M. (2019). An integrative model of cellular states, plasticity, and genetics for glioblastoma. Cell.

[28] Arvanitis C.D., Ferraro G.B., Jain R.K. (2020). The blood–brain barrier and blood–tumour barrier in brain tumours and metastases. Nat. Rev. Cancer.

[29] Gomolka R.S., Hablitz L.M., Mestre H., Giannetto M., Du T., Hauglund N.L., Xie L., Peng W., Martinez P.M., Nedergaard M. (2023). Loss of aquaporin-4 results in glymphatic system dysfunction via brain-wide interstitial fluid stagnation. eLife.

[30] Tan X., Li X., Li R., Meng W., Xie Z., Li J., Pang Y., Huang G., Li L., Li H. (2024). β-hydroxybutyrate alleviates neurological deficits by restoring glymphatic and inflammation after subarachnoid hemorrhage in mice. Exp. Neurol..

[31] Zhang H., Wang J., Zhang S., Yan D., Dong Y., Zhang P., Sun W., Liu X. (2025). Aquaporin 4 and its isoforms regulation ameliorate AQP4 Mis-localization-induced glymphatic dysfunction in ischemic stroke. J. Adv. Res..

[32] Simon M., Wang M.X., Ismail O., Braun M., Schindler A.G., Reemmer J., Wang Z., Haveliwala M.A., O’Boyle R.P., Han W.Y. (2022). Loss of perivascular aquaporin-4 localization impairs glymphatic exchange and promotes amyloid β plaque formation in mice. Alzheimer’s Res. Ther..

[33] Kaur B., Khwaja F.W., Severson E.A., Matheny S.L., Brat D.J., Van Meir E.G. (2005). Hypoxia and the hypoxia-inducible-factor pathway in glioma growth and angiogenesis. Neuro-Oncology.

[34] Rong Y., Durden D.L., Van Meir E.G., Brat D.J. (2006). ‘Pseudopalisading’necrosis in glioblastoma: A familiar morphologic feature that links vascular pathology, hypoxia, and angiogenesis. J. Neuropathol. Exp. Neurol..

[35] Hara T., Chanoch-Myers R., Mathewson N.D., Myskiw C., Atta L., Bussema L., Eichhorn S.W., Greenwald A.C., Kinker G.S., Rodman C. (2021). Interactions between cancer cells and immune cells drive transitions to mesenchymal-like states in glioblastoma. Cancer Cell.

[36] Xiao M., Marshall C., Yin K. (2025). Glymphatic-meningeal lymphatic system, a new therapeutic target for sleep disorders. Neuroscience.

[37] Reddy O.C., van der Werf Y.D. (2020). The sleeping brain: Harnessing the power of the glymphatic system through lifestyle choices. Brain Sci..

[38] Voumvourakis K.I., Sideri E., Papadimitropoulos G.N., Tsantzali I., Hewlett P., Kitsos D., Stefanou M., Bonakis A., Giannopoulos S., Tsivgoulis G. (2023). The dynamic relationship between the glymphatic system, aging, memory, and sleep. Biomedicines.

[39] Chong P.L., Garic D., Shen M.D., Lundgaard I., Schwichtenberg A.J. (2022). Sleep, cerebrospinal fluid, and the glymphatic system: A systematic review. Sleep Med. Rev..

[40] Iwadate Y. (2016). Epithelial-mesenchymal transition in glioblastoma progression. Oncol. Lett..

