# Supplementary material for: Integrated Transcriptomic Analysis Reveals Coordinated Gliovascular Remodeling Across Diffuse Glioma Grades
Source: Cancers (Basel). 2026 Sep 9;18(18):2908. doi: 10.3390/cancers18182908 (PMC13604490; doi:10.3390/cancers18182908)
Supplement: Supplementary file 1 [file cancers-18-02908-s001.zip › cancers-4530188-supplementary.pdf]

Supplementary Materials

Integrated Transcriptomic Analysis Reveals Coordinated Gliovascular Remodeling Across Diffuse Glioma Grade

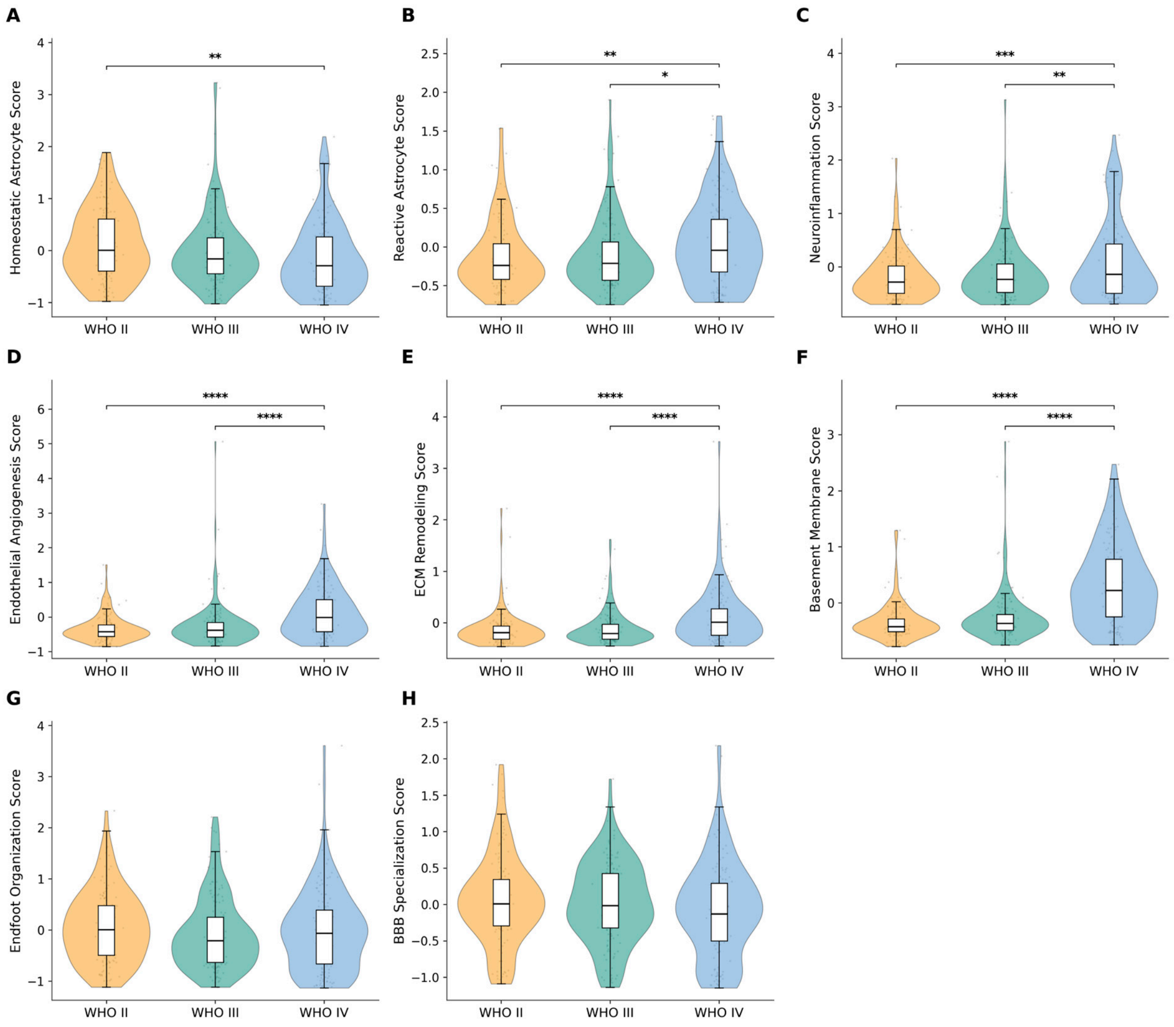

Figure S1. Pathway activity scores across WHO glioma grade, CGGA validation cohort. Violin plots of eight pathway activity scores (A–H: homeostatic astrocytes, reactive astrocytes, neuroinflammation, endothelial angiogenesis, ECM remodeling, basement membrane, endfoot organization, BBB specialization) stratified by WHO grade (II, III, IV) in the independent CGGA validation cohort (n = 422). Box plots show median and interquartile range; individual points show single samples. Significance was assessed using one-way ANOVA followed by Tukey's honestly significant difference (HSD) post-hoc test for pairwise comparisons between grades; only statistically significant pairwise comparisons are displayed (* $p < 0.05$, ** $p < 0.01$, *** $p < 0.001$, **** $p < 0.0001$).

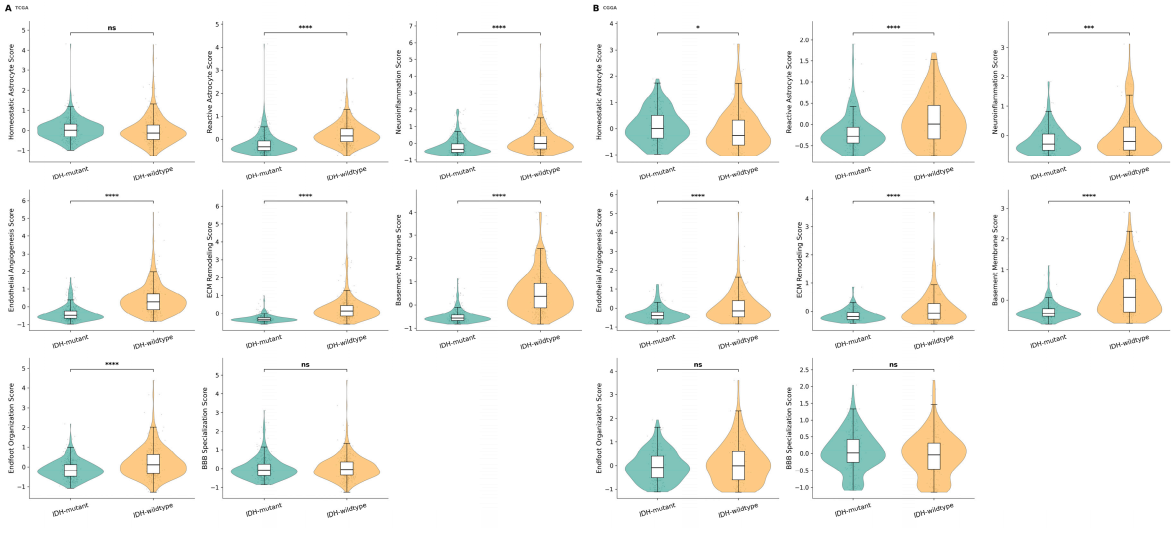

Figure S2. Validation of IDH-associated pathway scores, TCGA and CGGA. (A) TCGA and (B) CGGA: violin plots of the eight pathway scores by IDH mutation status (mutant vs. wildtype). Significance from Welch's t-test (* $p < 0.05$, *** $p < 0.001$, **** $p < 0.0001$; ns, not significant).

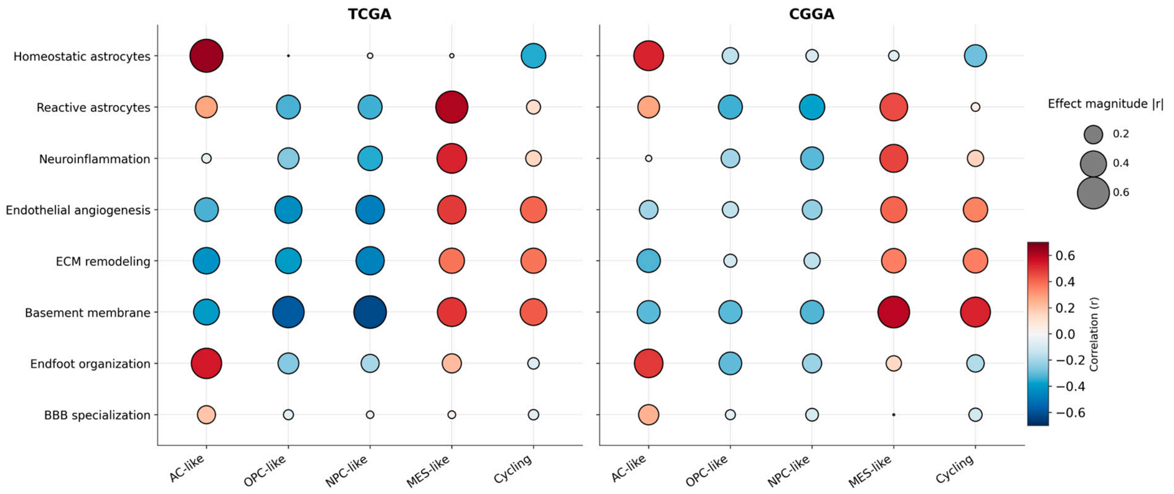

Figure S3. Pathway associations with Neftel-derived transcriptional programs. Bubble plot of Pearson correlations between the eight pathway scores and five malignant cell-state scores (AC-like, OPC-like, NPC-like, MES-like, cycling) described by Neftel et al., in TCGA and CGGA. Circle size indicates |r|; color indicates direction and magnitude.

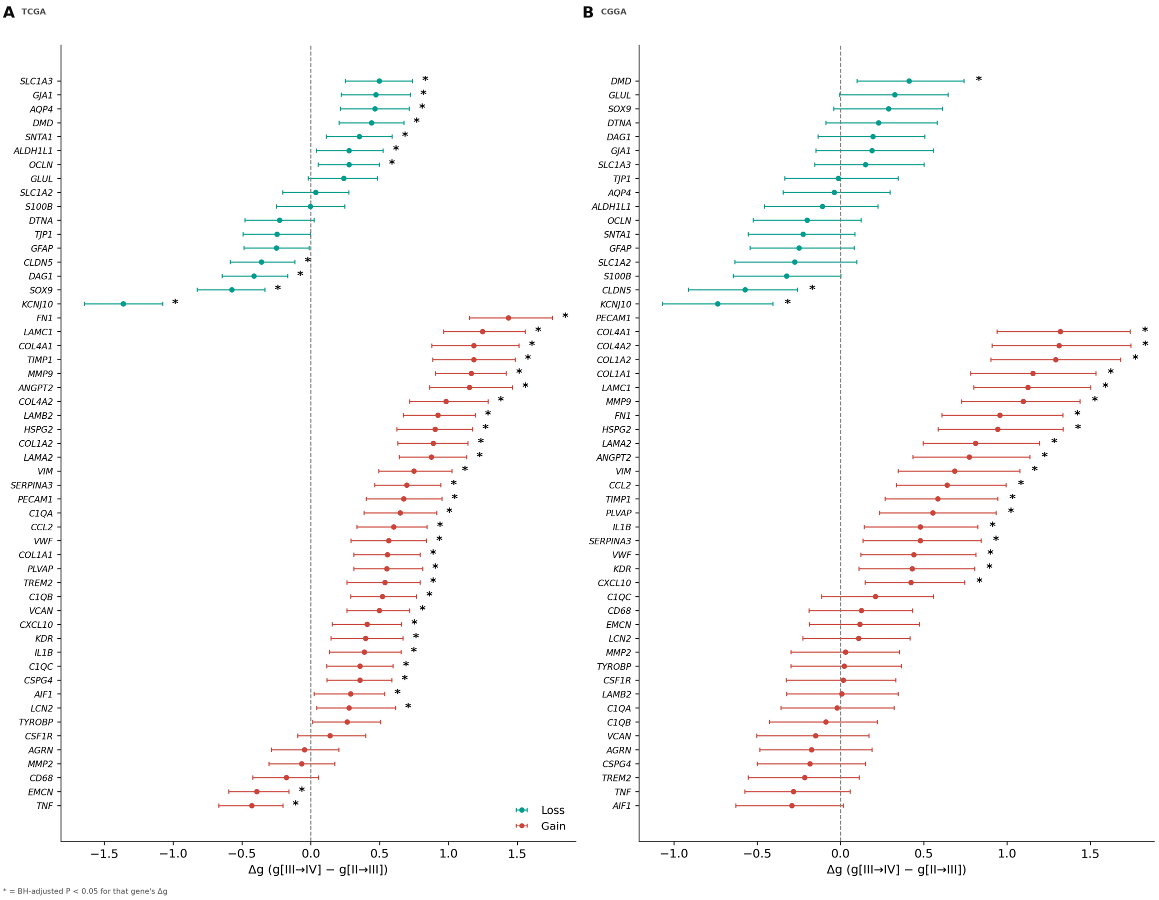

Figure S4. Per-gene grade-specific effect-size differences by Loss/Gain category. (A) TCGA and (B) CGGA: per-gene difference in grade-specific effect size (Δg = Hedges' g[WHO III–IV] − Hedges' g[WHO II–III], bootstrap 95% CI) for all 53 core-category genes (Loss, teal; Gain, red), individually. Asterisks indicate BH-adjusted $p < 0.05$ for that gene's Δg.

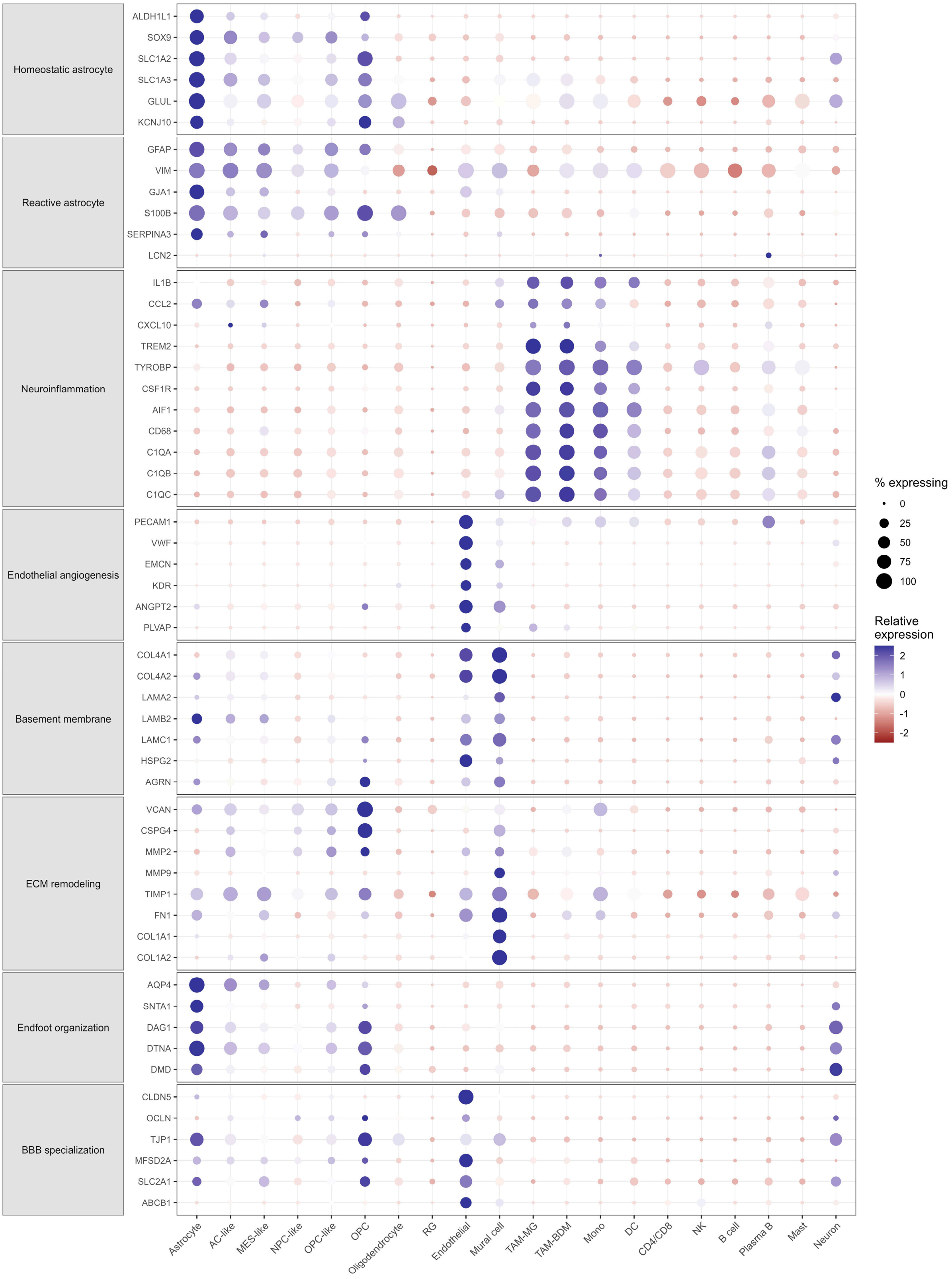

Homeostatic astrocyte
Reactive astrocyte
Neuroinflammation
Endothelial angiogenesis
Basement membrane
ECM remodeling
Endfoot organization
BBB specialization
ALDH1L1
SOX9
SLC1A2
SLC1A3
GLUL
KCNJ10
GFAP
VIM
GJA1
S100B
SERPINA3
LCN2
IL1B
CCL2
CXCL10
TREM2
TYROBP
CSF1R
AIF1
CD68
C1QA
C1QB
C1QC
PECAM1
VWF
EMCN
KDR
ANGPT2
PLVAP
COL4A1
COL4A2
LAMA2
LAMB2
LAMC1
HSPG2
AGRN
VCAN
CSPG4
MMP2
MMP9
TIMP1
FN1
COL1A1
COL1A2
AQP4
SNTA1
DAG1
DTNA
DMD
CLDN5
OCLN
TJP1
MFSD2A
SLC2A1
ABCB1
Astrocyte
AC-like
MES-like
NPC-like
OPC-like
OPC
Oligodendrocyte
RG
Endothelial
Mural cell
TAM-MG
TAM-BDM
Mono
DC
CD4/CD8
NK
B cell
Plasma B
Mast
Neuron
% expressing
0
25
50
75
100
Relative expression
2
1
0
-1
-2

Figure S5. Cell-type expression specificity of the 55 gliovascular marker genes, single-cell GBM reference atlas (338,564 cells). Dot plot showing the percentage of cells expressing each marker gene (dot size) and relative, row-scaled expression level (color) across annotated cell types, including malignant cell states (AC-like, MES-like, NPC-like, OPC-like), non-malignant glia (astrocyte, oligodendrocyte, oligodendrocyte precursor cell [OPC], radial glia [RG]), vascular cells (endothelial, mural), myeloid populations (microglia-derived and monocyte-derived tumor-associated macrophages [TAM-MG, TAM-BDM], monocytes [Mono], dendritic cells [DC]), lymphoid populations (CD4/CD8 T cells, natural killer [NK] cells, B cells, plasma B cells, mast cells), and neurons. Genes are grouped by their assigned pathway (Table 1).

Table S1. Sensitivity of grade-associated gliovascular pathway effects to alternative ssGSEA scoring

Cohort	Pathway	Primary β	Primary P	ssGSEA β	ssGSEA P	Direction concordant
TCGA	Endfoot organization	0.008	0.769	-0.005	0.019	No
TCGA	Homeostatic astrocytes	-0.202	1.87×10^{-14}	-0.024	1.28×10^{-15}	Yes
TCGA	Reactive astrocytes	0.153	8.25×10^{-13}	0.025	7.26×10^{-16}	Yes
TCGA	BBB specialization	0.004	0.876	-0.025	2.88×10^{-19}	No
TCGA	Endothelial angiogenesis	0.492	3.89×10^{-59}	0.072	4.61×10^{-72}	Yes
TCGA	Basement membrane	0.606	1.78×10^{-84}	0.133	$<1 \times 10^{-99}$	Yes
TCGA	ECM remodeling	0.368	7.68×10^{-53}	0.124	$<1 \times 10^{-99}$	Yes
TCGA	Neuroinflammation	0.245	2.27×10^{-17}	0.027	2.90×10^{-13}	Yes
CGGA	Endfoot organization	-0.043	0.330	-0.005	0.333	Yes
CGGA	Homeostatic astrocytes	-0.136	9.81×10^{-4}	-0.032	1.83×10^{-10}	Yes
CGGA	Reactive astrocytes	0.093	0.001	0.021	2.88×10^{-10}	Yes
CGGA	BBB specialization	-0.068	0.059	-0.012	0.004	Yes
CGGA	Endothelial angiogenesis	0.226	7.86×10^{-10}	0.086	1.59×10^{-25}	Yes
CGGA	Basement membrane	0.331	4.30×10^{-22}	0.112	2.21×10^{-33}	Yes
CGGA	ECM remodeling	0.128	2.16×10^{-7}	0.110	3.68×10^{-34}	Yes
CGGA	Neuroinflammation	0.136	1.13×10^{-4}	0.031	5.01×10^{-7}	Yes

Table S2. Leave-one-gene-out sensitivity analysis of grade-associated gliovascular pathway scores

Cohort	Pathway	Genes in full score	Full β	Full P	LOO β range	Direction preserved	All LOO significant
TCGA	Endfoot organization	5	0.008	0.769	-0.060 to 0.065	No	No
TCGA	Homeostatic astrocytes	6	-0.202	1.87 × 10^-14	-0.275 to -0.078	Yes	Yes
TCGA	Reactive astrocytes	6	0.153	8.25 × 10^-13	0.075 to 0.226	Yes	Yes
TCGA	BBB specialization	6	0.004	0.876	-0.067 to 0.088	No	No
TCGA	Endothelial angiogenesis	6	0.492	3.89 × 10^-59	0.444 to 0.567	Yes	Yes
TCGA	Basement membrane	7	0.606	1.78 × 10^-84	0.585 to 0.657	Yes	Yes
TCGA	ECM remodeling	8	0.368	7.68 × 10^-53	0.348 to 0.390	Yes	Yes
TCGA	Neuroinflammation	12	0.245	2.27 × 10^-17	0.226 to 0.282	Yes	Yes
CGGA	Endfoot organization	5	-0.043	0.330	-0.079 to -0.017	Yes	No
CGGA	Homeostatic astrocytes	6	-0.136	9.81 × 10^-4	-0.184 to -0.081	Yes	Yes
CGGA	Reactive astrocytes	6	0.093	0.001	0.038 to 0.134	Yes	No
CGGA	BBB specialization	6	-0.068	0.059	-0.110 to -0.031	Yes	No
CGGA	Endothelial angiogenesis	5	0.226	7.86 × 10^-10	0.193 to 0.260	Yes	Yes
CGGA	Basement membrane	7	0.331	4.30 × 10^-22	0.313 to 0.353	Yes	Yes
CGGA	ECM remodeling	8	0.128	2.16 × 10^-7	0.095 to 0.178	Yes	Yes
CGGA	Neuroinflammation	12	0.136	1.13 × 10^-4	0.125 to 0.161	Yes	Yes

Table S3. Sensitivity analysis of associations between gliovascular signatures and Neftel-derived transcriptional programs after removal of overlapping genes

*For each gliovascular pathway – Neftel program pair with shared marker genes, the correlation (Pearson r) is shown before and after excluding genes counted in both gene sets, in TCGA and CGGA. *Gene overlaps with the corresponding Neftel meta-module and was removed from the gliovascular pathway signature for the sensitivity analysis. †After removal of overlapping genes, the reactive astrocyte-associated signature contained only LCN2 (1 of 6 genes); therefore, the resulting correlation should be interpreted cautiously because it no longer represents a multigene pathway signature. ‡After removal of overlapping genes, the ECM-remodeling signature contained 5 of the original 8 genes.*

Gliovascular signature	Neftel program	Shared genes removed	Genes remaining	TCGA r original → excluded	CGGA r original → excluded
Homeostatic astrocyte	AC-like	SLC1A3	5/6	0.641 → 0.719	0.524 → 0.547
Reactive astrocyte	AC-like	GFAP, S100B*	1/6†	0.274 → −0.109	0.279 → −0.169
Reactive astrocyte	MES-like	VIM, GJA1, SERPINA3*	1/6†	0.607 → 0.146	0.452 → 0.096
ECM remodeling	OPC-like	VCAN*	5/8‡	−0.390 → −0.461	−0.100 → −0.204
ECM remodeling	MES-like	TIMP1, FN1*	5/8‡	0.375 → 0.617	0.352 → 0.648
Endfoot organization	AC-like	AQP4	4/5	0.544 → 0.611	0.482 → 0.388
BBB specialization	MES-like	SLC2A1	5/6	0.034 → −0.085	0.002 → −0.090

Table S4. Mixed-effects model results for regional expression of representative gliovascular genes in Ivy GAP

Panel A. Omnibus regional effects and variance components

Gene	n	Tumors	Num df	Den df	F	P	Tumor variance	Residual variance
KCNJ10	122	10	4	109.27	101.51	6.99×10^{-36}	0.3108	0.3805
SNTA1	122	10	4	110.72	76.74	5.00×10^{-31}	0.1301	0.3727
AQP4	122	10	4	109.00	60.06	1.06×10^{-26}	0.6388	0.9341
SERPINA3	122	10	4	109.82	21.36	4.73×10^{-13}	1.4387	2.4453
CCL2	122	10	4	109.05	14.22	2.26×10^{-9}	1.9526	1.8795
FN1	122	10	4	110.46	163.61	1.86×10^{-45}	0.2027	0.7081
COL4A1	122	10	4	110.94	218.33	1.31×10^{-51}	0.1954	0.8548
ANGPT2	122	10	4	111.80	94.06	7.38×10^{-35}	0.1629	1.2054
TJP1	122	10	4	109.40	29.05	1.87×10^{-16}	0.1296	0.2178

Panel B. Fixed-effect coefficients (LE reference)

Gene	Term	Estimate	SE	df	t	P
KCNJ10	Intercept	3.8101	0.2293	20.22	16.618	2.93×10^{-13}
KCNJ10	IT vs LE	-0.4405	0.1907	108.28	-2.310	0.0228
KCNJ10	CT vs LE	-0.8305	0.1849	109.42	-4.492	1.75×10^{-5}
KCNJ10	MVP vs LE	-2.8476	0.1910	108.86	-14.913	4.59×10^{-28}
KCNJ10	PAN vs LE	-2.7281	0.1983	110.61	-13.760	1.04×10^{-25}
SNTA1	Intercept	4.8539	0.1840	35.68	26.387	5.30×10^{-25}
SNTA1	IT vs LE	-0.7725	0.1886	109.13	-4.096	8.10×10^{-5}
SNTA1	CT vs LE	-1.3327	0.1824	110.98	-7.308	4.43×10^{-11}
SNTA1	MVP vs LE	-3.0806	0.1886	110.16	-16.334	3.36×10^{-31}
SNTA1	PAN vs LE	-1.6195	0.1950	112.92	-8.306	2.43×10^{-13}
AQP4	Intercept	8.6486	0.3414	21.27	25.333	2.25×10^{-17}
AQP4	IT vs LE	0.3398	0.2988	107.82	1.137	0.2579
AQP4	CT vs LE	-0.5107	0.2895	109.18	-1.764	0.0805
AQP4	MVP vs LE	-3.0130	0.2991	108.52	-10.074	3.02×10^{-17}
AQP4	PAN vs LE	-2.7234	0.3103	110.61	-8.776	2.37×10^{-14}
SERPINA3	Intercept	5.5327	0.5306	25.07	10.428	1.33×10^{-10}
SERPINA3	IT vs LE	3.8867	0.4833	108.61	8.042	1.18×10^{-12}
SERPINA3	CT vs LE	3.3155	0.4681	110.01	7.082	1.41×10^{-10}
SERPINA3	MVP vs LE	1.3950	0.4838	109.34	2.884	0.0047
SERPINA3	PAN vs LE	2.3867	0.5016	111.48	4.758	5.90×10^{-6}
CCL2	Intercept	3.7177	0.5492	17.82	6.769	2.56×10^{-6}
CCL2	IT vs LE	2.3335	0.4239	108.23	5.505	2.50×10^{-7}
CCL2	CT vs LE	1.9766	0.4112	109.18	4.807	4.92×10^{-6}
CCL2	MVP vs LE	2.6536	0.4246	108.70	6.250	8.22×10^{-9}
CCL2	PAN vs LE	3.0193	0.4412	110.16	6.843	4.58×10^{-10}
FN1	Intercept	2.2306	0.2443	37.92	9.129	4.12×10^{-11}
FN1	IT vs LE	1.4270	0.2599	108.56	5.490	2.66×10^{-7}
FN1	CT vs LE	1.6040	0.2511	110.76	6.388	4.07×10^{-9}
FN1	MVP vs LE	5.9999	0.2598	109.83	23.095	5.82×10^{-44}
FN1	PAN vs LE	2.5522	0.2682	113.10	9.516	3.98×10^{-16}
COL4A1	Intercept	1.2616	0.2588	43.98	4.875	1.45×10^{-5}
COL4A1	IT vs LE	2.3271	0.2855	108.89	8.151	6.65×10^{-13}
COL4A1	CT vs LE	3.5434	0.2755	111.26	12.861	9.53×10^{-24}
COL4A1	MVP vs LE	7.9027	0.2852	110.31	27.710	1.64×10^{-51}
COL4A1	PAN vs LE	2.7149	0.2939	113.78	9.237	1.69×10^{-15}
ANGPT2	Intercept	2.3222	0.2872	57.95	8.086	4.42×10^{-11}
ANGPT2	IT vs LE	1.6987	0.3387	109.45	5.015	2.06×10^{-6}
ANGPT2	CT vs LE	2.0062	0.3261	112.17	6.151	1.21×10^{-8}
ANGPT2	MVP vs LE	5.7707	0.3379	111.24	17.078	7.22×10^{-33}
ANGPT2	PAN vs LE	0.8385	0.3468	115.09	2.418	0.0172
TJP1	Intercept	4.7437	0.1588	23.77	29.872	2.33×10^{-20}

Gene	Term	Estimate	SE	df	t	P
TJP1	IT vs LE	0.1455	0.1442	108.14	1.009	0.3154
TJP1	CT vs LE	0.1117	0.1397	109.60	0.800	0.4256
TJP1	MVP vs LE	0.6432	0.1444	108.90	4.455	2.04×10^{-5}
TJP1	PAN vs LE	-0.8120	0.1497	111.13	-5.424	3.44×10^{-7}

Table S5. Pairwise regional comparisons for representative gliovascular genes, Ivy GAP

Gene	P < 0.0001 (consolidated)	Other comparisons (estimate, Tukey-adjusted P)
KCNJ10	LE - PAN; LE - MVP; IT - PAN; IT - MVP; CT - PAN; CT - MVP	LE - IT (0.44, P=0.1499); **LE - CT (0.83, P=0.0002)**; IT - CT (0.39, P=0.1669); PAN - MVP (0.12, P=0.9653)
SERPINA3	LE - IT; LE - CT; LE - PAN; IT - MVP	**LE - MVP (-1.40, P=0.0377)**; IT - CT (0.57, P=0.6887); **IT - PAN (1.50, P=0.0158)**; CT - PAN (0.93, P=0.2115); **CT - MVP (1.92, P=0.0002)**; PAN - MVP (0.99, P=0.2078)
SNTA1	LE - CT; LE - PAN; LE - MVP; IT - PAN; IT - MVP; CT - MVP; PAN - MVP	**LE - IT (0.77, P=0.0008)**; **IT - CT (0.56, P=0.0118)**; CT - PAN (0.29, P=0.4429)
AQP4	LE - PAN; LE - MVP; IT - PAN; IT - MVP; CT - PAN; CT - MVP	LE - IT (-0.34, P=0.7865); LE - CT (0.51, P=0.4016); **IT - CT (0.85, P=0.0180)**; PAN - MVP (0.29, P=0.8483)
CCL2	LE - IT; LE - CT; LE - PAN; LE - MVP	IT - CT (0.36, P=0.8850); IT - PAN (-0.69, P=0.4643); IT - MVP (-0.32, P=0.9285); CT - PAN (-1.04, P=0.0550); CT - MVP (-0.68, P=0.3805); PAN - MVP (0.37, P=0.8956)
TJP1	LE - PAN; IT - PAN; CT - PAN; PAN - MVP	LE - IT (-0.15, P=0.8510); LE - CT (-0.11, P=0.9306); **LE - MVP (-0.64, P=0.0002)**; IT - CT (0.03, P=0.9990); **IT - MVP (-0.50, P=0.0033)**; **CT - MVP (-0.53, P=0.0006)**
COL4A1	LE - IT; LE - CT; LE - PAN; LE - MVP; IT - CT; IT - MVP; CT - MVP; PAN - MVP	IT - PAN (-0.39, P=0.6285); **CT - PAN (0.83, P=0.0138)**
FN1	LE - IT; LE - CT; LE - PAN; LE - MVP; IT - MVP; CT - MVP; PAN - MVP	IT - CT (-0.18, P=0.9432); **IT - PAN (-1.13, P=0.0002)**; **CT - PAN (-0.95, P=0.0009)**
ANGPT2	LE - IT; LE - CT; LE - MVP; IT - MVP; CT - MVP; PAN - MVP	LE - PAN (-0.84, P=0.1235); IT - CT (-0.31, P=0.8519); IT - PAN (0.86, P=0.0719); **CT - PAN (1.17, P=0.0019)**

Table S6. Pairwise Spearman correlations among gliovascular pathway scores

A. TCGA

Pathway	Endfoot	Homeostatic	Reactive	BBB	Endothelial	Basement	ECM	Neuroinflam.
**Endfoot**	1.000	0.607	0.524	0.385	0.029	0.093	-0.065	0.073
**Homeostatic**	0.607	1.000	0.200	0.316	-0.283	-0.298	-0.377	-0.160
**Reactive**	0.524	0.200	1.000	0.107	0.273	0.358	0.242	0.594
**BBB**	0.385	0.316	0.107	1.000	0.343	0.122	0.018	-0.073
**Endothelial**	0.029	-0.283	0.273	0.343	1.000	0.780	0.688	0.354
**Basement**	0.093	-0.298	0.358	0.122	0.780	1.000	0.799	0.370
**ECM**	-0.065	-0.377	0.242	0.018	0.688	0.799	1.000	0.319
**Neuroinflam.**	0.073	-0.160	0.594	-0.073	0.354	0.370	0.319	1.000

B. CGGA

Pathway	Endfoot	Homeostatic	Reactive	BBB	Endothelial	Basement	ECM	Neuroinflam.
**Endfoot**	1.000	0.839	0.588	0.646	0.383	0.179	0.251	0.349
**Homeostatic**	0.839	1.000	0.438	0.684	0.292	-0.014	0.111	0.191
**Reactive**	0.588	0.438	1.000	0.448	0.487	0.496	0.330	0.676
**BBB**	0.646	0.684	0.448	1.000	0.591	0.240	0.298	0.339
**Endothelial**	0.383	0.292	0.487	0.591	1.000	0.694	0.612	0.463
**Basement**	0.179	-0.014	0.496	0.240	0.694	1.000	0.589	0.505
**ECM**	0.251	0.111	0.330	0.298	0.612	0.589	1.000	0.319
**Neuroinflam.**	0.349	0.191	0.676	0.339	0.463	0.505	0.319	1.000

Table S7. Robustness of core-chain partial correlations to tumor-purity adjustment, TCGA

Edge	r (grade-adjusted)	r (grade+purity-adjusted)	BH-P (purity-adj.)	% change
Homeostatic Astrocytes → Reactive Astrocytes	0.282	0.254	3.78e-11	+9.9%
Reactive Astrocytes → Neuroinflammation	0.372	0.334	5.88e-19	+10.4%
Neuroinflammation → ECM Remodeling	0.249	0.140	4.13e-04	+43.7%
ECM Remodeling → Basement Membrane	0.564	0.543	1.14e-55	+3.8%
Basement Membrane → Endothelial Angiogenesis	0.628	0.624	6.52e-80	+0.7%
